# Professional standards in bibliometric research evaluation? A meta-evaluation of European assessment practice 2005–2019

**DOI:** 10.1371/journal.pone.0231735

**Published:** 2020-04-20

**Authors:** Arlette Jappe

**Affiliations:** Interdisciplinary Centre of Science and Technology Studies (IZWT), University of Wuppertal, Wuppertal, Germany; Max Planck Society, GERMANY

## Abstract

Despite growing demand for practicable methods of research evaluation, the use of bibliometric indicators remains controversial. This paper examines performance assessment practice in Europe—first, identifying the most commonly used bibliometric methods and, second, identifying the actors who have defined wide-spread practices. The framework of this investigation is Abbott’s theory of professions, and I argue that indicator-based research assessment constitutes a potential jurisdiction for both individual experts and expert organizations. This investigation was conducted using a search methodology that yielded 138 evaluation studies from 21 EU countries, covering the period 2005 to 2019. Structured content analysis revealed the following findings: (1) Bibliometric research assessment is most frequently performed in the Nordic countries, the Netherlands, Italy, and the United Kingdom. (2) The Web of Science (WoS) is the dominant database used for public research assessment in Europe. (3) Expert organizations invest in the improvement of WoS citation data, and set technical standards with regards to data quality. (4) Citation impact is most frequently assessed with reference to international scientific fields. (5) The WoS classification of science fields retained its function as a de facto reference standard for research performance assessment. A detailed comparison of assessment practices between five dedicated organizations and other individual bibliometric experts suggests that corporate ownership and limited access to the most widely used citation databases have had a restraining effect on the development and diffusion of professional bibliometric methods during this period.

## 1. Introduction

Research organizations and research funding agencies have a growing demand for practicable methods of research evaluation, including metrics based on publication and citation data. Such bibliometric indicators remain controversial among the scientific communities affected by performance assessment [1–5]. In recent years, several studies have reviewed scientific developments in the area of evaluative citation analyses [6–11]. However, there is little overview regarding which bibliometric methods are actually applied by practitioners in research evaluation. This gap is addressed by the present paper. In the literature, the expression ‘meta-evaluation’ is commonly used to denote systematic reviews of evaluation studies with regards to methodological quality and results [12–14]. Similarly, an ‘evaluation synthesis’ reviews the findings of an already existing set of evaluations [15]. In the present study, I analyse the methodologies of existing evaluation studies from a meta-perspective. However, rather than evaluating published studies according to predefined methodological standards of good practice, my purpose here is to investigate two main research questions. First, what were the prevailing methodological standards, referred to as professional de facto standards, in the field of research assessment practice during a certain period? Second, if certain de facto standards of bibliometric research assessment can be identified, which social actors have defined them?

The methodological focus of this study is on the measurement of citation impact. Other topics of bibliometric assessment, such as emerging research topics, research profiles, international collaboration are excluded. Research productivity in the sense of an input–output relationship is only assessed in a few instances, since adequate data on resource inputs (scientific staff and funding streams) are often not available [16]. Moreover, this investigation only includes ‘real’ evaluations, i.e. assessments conducted for purposes of decision-making in research policy or research management. I applied several complementary search strategies, and identified 138 individual studies published during the period 2005–2019, which evaluated either research organizations (RO) or research funding instruments (FI) from 21 European countries plus EU framework programs. This study gives an overview on professional practices during fifteen years of expansion of bibliometric research assessment. My initial assumption was that leading organizations within the expert field would be influential in defining professional de facto standards—first, because they have a high market share of assessment services and, second, because they serve as a legitimate role model that is imitated by other bibliometric experts. The findings support this assumption but also highlight the importance of data access and data distribution for the operative establishment of de facto standards. All studies are documented in the Annex (Supplementary Tables).

This paper is part of a project conducted with the aim of understanding the development of bibliometric assessment methods from the perspective of Abbott’s sociological theory of professions [17, 18]. I selected this theory to investigate how particular methodological choices become socially established as professionally legitimate means of handling certain evaluation problems. More specifically, this framework is used to address the issue of professional control in bibliometric assessment. Applying Abbott’s terminology, the increasing demand for practicable and efficient assessment of academic performance constitutes a problem amenable to expert service. Research assessment is potentially within the jurisdiction of professional experts who can define the nature of assessment problems, and offer solutions that effectively address clients’ needs. One recent paper presents an empirical investigation of whether the academic research area of ‘evaluative citation analysis’ has successfully defined scientific standards for bibliometric research evaluation during the period 1972–2016 [19]. Based on organizational network analysis and the theory of intellectual fields as reputational organizations, [19] concluded that the field of evaluative citation analysis has been characterized by low levels of reputational control, evidenced by high shares of outsider contributions and new actors entering the field throughout the examined period. They argued that this lack of reputational control within the academic research area is consistent with observed difficulties in establishing scientific authority for bibliometric assessment practice.

In this paper, I first present theoretical considerations concerning the application of professional sociology to the field of bibliometric research evaluation. Then I describe data and methods of meta-evaluation, including search strategies, selection criteria, and the content analysis of evaluation studies with regards to their methodological design and metrics. Finally, I present empirical findings and discuss results in light of the theoretical framework.

## 2. Theoretical considerations

**Fig 1:**
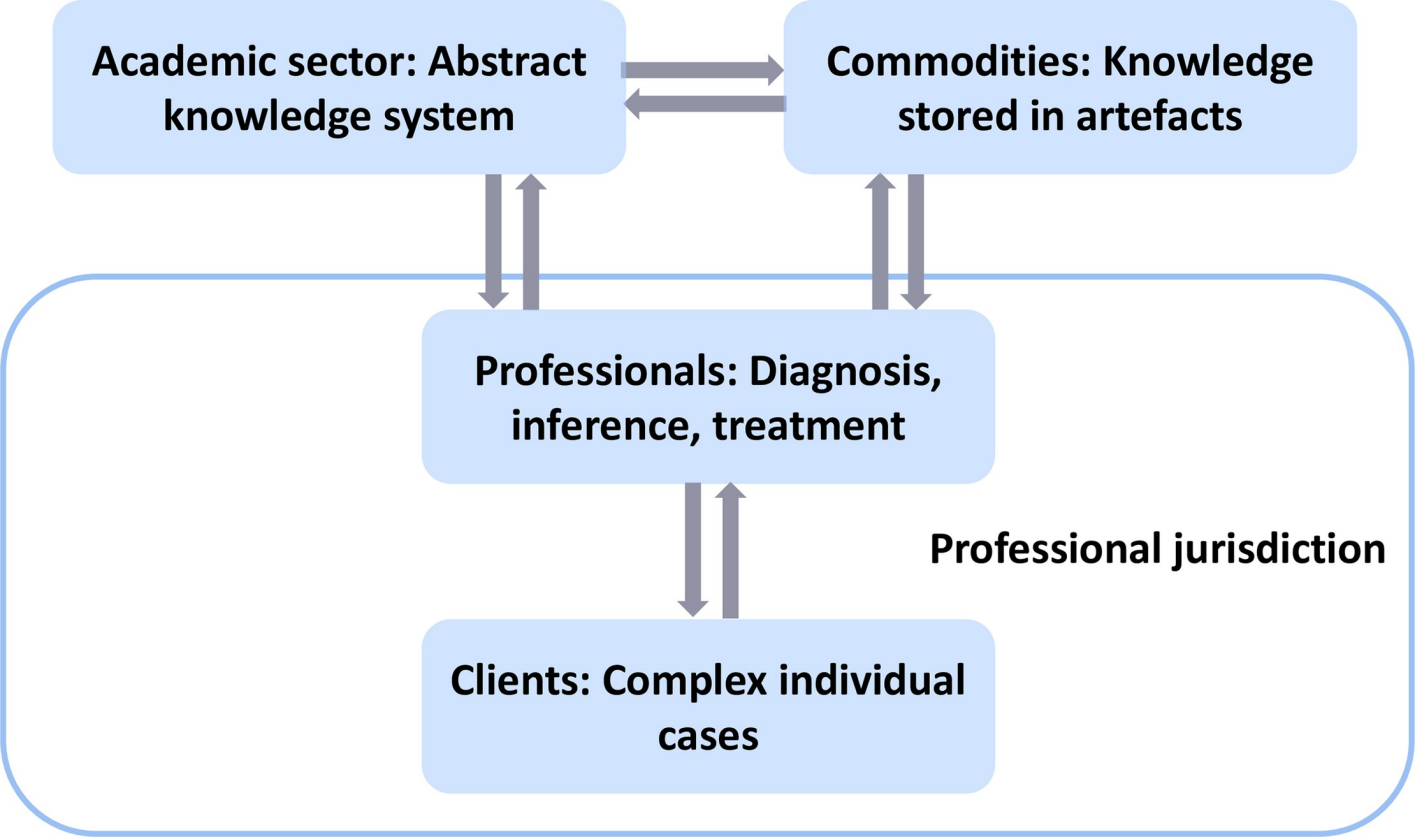
Theoretical framework of a mature profession according to Andrew Abbott. Visualization of the theoretical framework of a professional jurisdiction [17, 18]. Source: [19].

Abbott’s theory of professions is a sociological framework for analysing how professional expertise is socially constructed and institutionalized in modern societies [17, 18] (Fig 1). It is applicable in the setting of a societal problem amenable to expert service, and where groups of professional actors claim relevant expertise for treatment of this problem. The theory distinguishes between cognitive claims of expert knowledge versus social claims for jurisdiction with regards to the problem diagnosis and treatment that professionals must establish in various social arenas—including the legal system, the public, and the workplace. The concept of professional jurisdiction extends beyond a merely economic notion (i.e. a market for expert services) by including the potential development of expert control regarding appropriate problem definitions and treatments from a socio-historical perspective. This framework is suitable for cross-national comparisons since it makes no specific theoretical assumptions concerning the nation state’s role in the eventual settlement of professional jurisdictions. I selected this conceptual framework for the present project because it is suitable for investigating emerging professions that lack recognized domains of expertise, and which may eventually be protected by state licences but are currently engaged in competition with other professional actors for the appropriation of relatively new jurisdictions or tasks.

**Fig 2:**
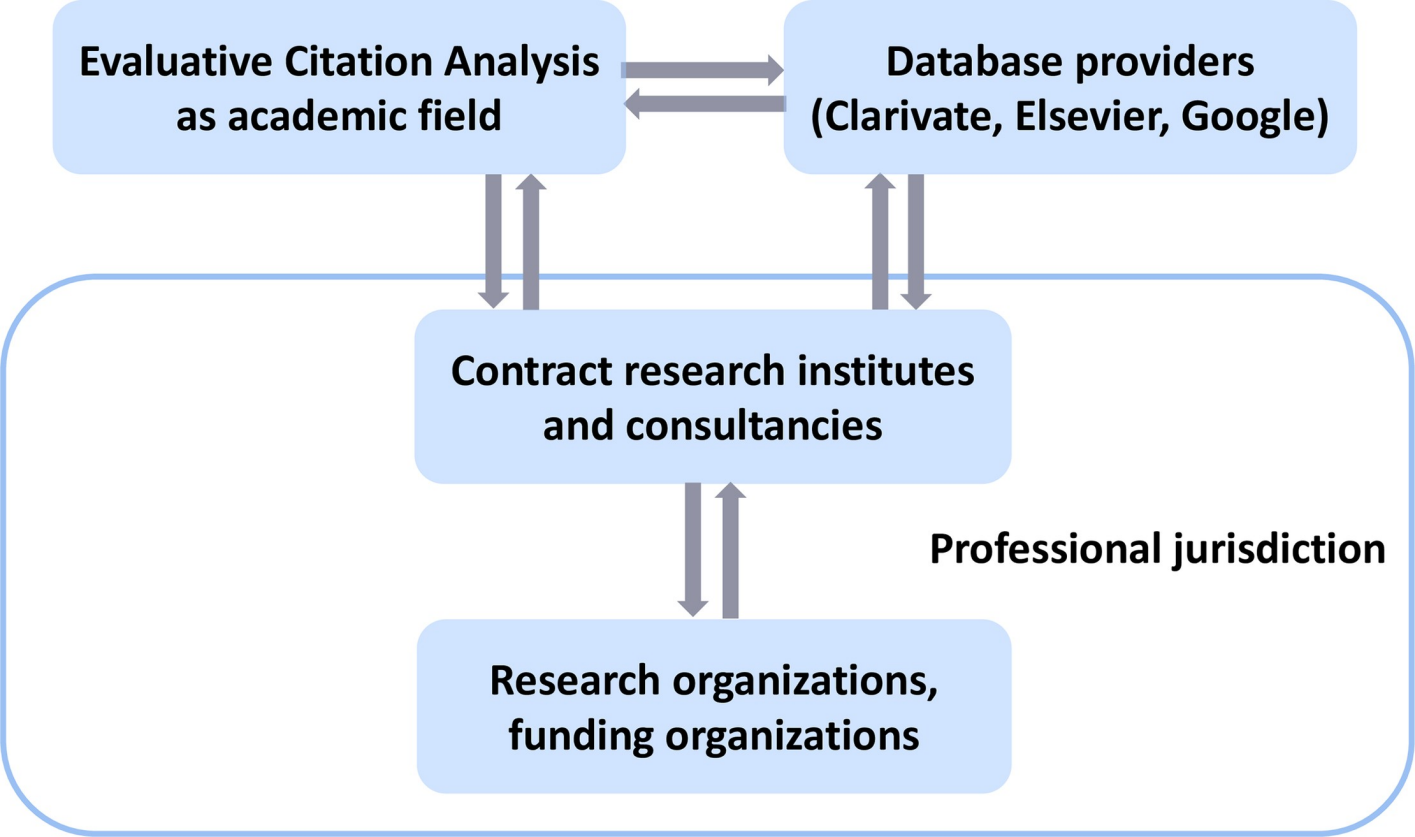
Bibliometric research assessment as an emerging profession. Visualization of the application of the theoretical framework in Fig 1 to the professional field of bibliometric research assessment. Source: [19].

In applying this theoretical framework to the realm of quantitative research assessment, I assume that the demand for professional services predominantly arises from two important groups of potential clients: research organizations and research funding agencies (Fig 2). These organizations require reliable information concerning the performance of their scientists, research groups, and funded projects for decision-making purposes [20, 21], and for accountability and legitimacy [22, 23]. I thus assume that demand largely stems from a meso-level of organizations within the public research system. Although private firms also use bibliometrics, information concerning research performance assessment in the private sector is not systematically accessible and is thus not part of the present study. Additionally, several European countries are experimenting with the introduction of performance metrics to evaluate institutional funding and research on a national scale [24–29]. Italy demonstrated the most extensive use of bibliometric performance measurement during the observation period. In 2006, the Italian National Agency for the Evaluation of Universities and Research Institutes (ANVUR) was created with the mandate to evaluate all public research—an exercise called “Valutazione della Qualità della Ricerca” (VQR) [24]. The present meta-evaluation included bibliometric reports from first two rounds of the VQR, covering the 2004–2010 (VQR I) and 2011–2014 (VQR II) [30], as well as national evaluations with a disciplinary scope conducted by the Nordic Institute for Studies in Innovation, Research and Education (NIFU). Since the 7th framework programme, and continued under Horizons 2020, the European Commission implemented the “Research infrastructure for research and innovation policy studies” (RISIS) with the aim to “build a distributed infrastructure on data relevant for research and innovation dynamics and policies”, but this collaborative project does not include the development of alternative literature and citation databases [31].

According to the theoretical framework, bibliometric assessment is a service provided by professionals. In 90% of studies in this meta-evaluation, bibliometric analyses were conducted by external experts, who often worked on behalf of the organization to be evaluated. Another 9% of studies were performed by bibliometricians employed by large non-university research organizations and funding agencies—namely the Spanish Consejo Superior de Investigaciones Científicas (CSIC), the German Max Planck Society (MPG), and the Swedish Research Council (VR).

I distinguish between individual bibliometric experts and organizations that are dedicated to bibliometric assessment services. Individual bibliometric experts are typically academics employed at universities or non-university research organizations, who conduct bibliometric studies as part of their individual research activities. The label “dedicated organizations” includes the Dutch Centre for Science and Technology Studies CWTS; the Nordic Institute for Studies in Innovation, Research, and Education NIFU in Oslo; the consultancy branch of the Web of Science Group owned by Clarivate Analytics, abbreviated here as TR/ Clarivate; ANVUR, the Italian state agency that implements the VQR; and the expert group working at CSIC, a large Spanish non-university research organization. CWTS, NIFU, and TR/ Clarivate are also referred to in the text as “expert organizations” in the sense of [18], while ANVUR is a state agency, and the CSIC bibliometric group conducts only studies on CSIC and its branches and do not offer professional services to other clients.

According to Abbott’s theory, professionals’ work can generally be described as the application of abstract knowledge to complex individual cases. Abstract knowledge lends legitimacy to claims of jurisdiction, tying professional work to the general values of logical consistency, rationality, effectiveness, and progress. Such scientific legitimacy includes definition of the nature of problems, a rational means of diagnosing these problems, and delivery of effective treatment. Moreover, abstract knowledge enables the instruction and training of students entering the profession, and facilitates the generation of new mechanisms of diagnosis, inference, and treatment. Abstract knowledge is typically accumulated by an academic sector closely related to the profession.

In a recent study, [19] investigated the academic research area of evaluative citation analysis as the academic sector that is closely aligned with bibliometric evaluation practice. Abstract knowledge can also be stored in specialized artefacts, which Abbott refers to as expert commodities. With regards to evaluative bibliometrics, the most important artefacts for professional work are citation databases, such as Web of Science (WoS) or Scopus. Another recent study [32] showed how science policy in the Netherlands stimulated the formation of quantitative research assessment as a new professional jurisdiction since the late 1960s in the form of an expert organization: CWTS. Using Abbott’s framework, this study argues that the professional work of CWTS is subordinate to the older jurisdiction of peer review and may develop into an advisory jurisdiction in the future.

The present study complements the two aforementioned studies and examines actual evaluation practice, as visible in mostly publicly available evaluation studies. The included studies use publication and citation data to evaluate the performance of research organizations or funding instruments in Europe, and are published either as study reports (grey literature) or as journal articles (see methods). The authors of these studies include bibliometric experts and dedicated organizations, while the objects of evaluation are research organizations and funding instruments. The presently utilized definition of professional practice excludes ad-hoc uses of bibliometric indicators that are less explicitly codified—for example, in cases where research organizations use performance metrics to evaluate staff performance, or where funding agencies use a journal impact factor or the h-index to make unpublished selection decisions among program applicants. This study is confined to Europe, i.e. the evaluation objects must be located in a European country. Thus, my analysis of widespread assessment practices further contributes to the knowledge of commonalities in a European research area, as promoted by the European Commission [33].

### 3. Data and methods

In this section, I first describe the selection criteria for inclusion of studies, as well as the search strategies used to identify such studies, and discuss generalizability in light of this sampling strategy. Second, I present the coding scheme and procedures applied to extract methodological information from each individual study. In line with the theoretical framework, practices of bibliometric research evaluation are examined for an extended historical time period. The study focuses on work products of professional actors in the form of published evaluation reports (grey literature) or journal articles. In most cases, these evaluation studies have been designed by bibliometric experts for their individual clients, i.e. decision makers from research organizations or funding agencies, and the professional diagnostics proposed by the bibliometricians have been accepted by the respective clients in a contract relationship. Therefore, assessment reports document an important segment of professional work. Other, less codified applications of bibliometric indicators were excluded, in particular performance assessment by private firms and research laboratories, software tools such as SciVal and Incites, and unpublished or ad-hoc use for decision-making by research organizations or funding agencies. While these uncodified applications also seem relevant for the question of de-facto standards, there is currently no systematic information on their usage and diffusion. The selection criteria for professional evaluation studies were as follows:

1. Each evaluation must include a publication and citation analysis. The sample includes studies relying exclusively on bibliometric data, as well as multi-dimensional evaluations that combine bibliometric data with other information, such as peer evaluations, financial and staff data, or case studies [34, 35]. In either scenario, my analysis focused only on bibliometric analyses.
2. The objects of evaluation are either research organizations or funding instruments. Research organizations are typically universities and/or their departments or faculties, or extra-university public research institutes. Funding instruments are typically active in supporting research projects or individual researchers at public research organizations, sometimes with the involvement of private firms, and sometimes supporting long-term investments, such as excellence schemes.
3. Evaluation objects (research organizations and funding instruments) must be located in Europe.
4. The evaluation had to be conducted with the stated purpose of informing decision making on behalf of the respective research organizations or funding instrument. Purely academic studies of bibliometric data were excluded from the study sample.
5. All analysed evaluations, including grey literature (project reports) and journal articles, were published between 2005 and 2019.

Sampling started in 2015, resulting in 85 evaluation studies for the period 2005–2014. In November/December 2019, sampling was updated, resulting in 53 additional studies for the period 2015–2019. I combined the following search strategies in order to identify a maximum number of studies from diverse sources:

1. Expert organizations and individual experts having a central position in the academic research area of evaluative citation analysis were identified by prior research [19]. Requests for non-confidential evaluation studies were sent in early 2015 to experts from 35 research organizations in 13 European countries. I received responses from 18 organizations (51%), of which 11 (31%) shared evaluation reports or information about published studies. A total of 16 studies (12% of the eventual sample) were identified in this way.
2. Evaluation studies were extracted from a set of WoS publications identified as “follow-up research on citation impact indicators” [19]. This set comprised all publications in WoS that cite any of 169 specified citation impact indicators, including a total of 2757 publications from 2005–2014 and 2776 publications from 2015–2019. Relevant studies from this sample were identified by analysing keywords and journal titles. The most frequent keywords related to research evaluation were “performance”, “universities”, “departments”, and “faculty”. I identified ten relevant journals within this publication sample, including “Research Evaluation”, “Research Policy”, “Research in Higher Education”, “Evaluation Review”, “Zeitschrift für Evaluation”, and “Higher Education”. From an initial set of 898 publications that were identified as potentially relevant (315 for 2005–2014; 583 for 2015–2019), 33 evaluation studies were retained (24% of the study sample). This search strategy proved particularly valuable in that it retrieved a total of eleven assessments published in medical journals, including for example the *British Journal of Neurosurgery* or the *European Journal of Cancer*, and four other disciplinary and foreign language journals, that would have escaped a more conventional search strategy based on a pre-defined set of core journals in scientometrics, science policy, and research evaluation.
3. I searched for bibliometric studies in the “Science and Innovation Policy Evaluation Repository” (SIPER), which was created under the 7^th^ framework program of the European Union. This publicly accessible database contains meta-data, as well as original documents of evaluation studies [36]. A search for citation analysis retrieved 32 potentially relevant documents, six of which were retained (4% of study sample).
4. I used material from [32], including 24 evaluation reports by CWTS, and three Dutch evaluations by other bibliometric authors (2%) for the period 2005–2014. For the period 2015–2019, additional CWTS reports were coded at a visit at CWTS Leiden, plus some published in the internet, resulting in 37 reports for CWTS in the total sample (27% of study sample).
5. I included reports from the Italian national evaluation agency ANVUR which has a legal mandate to evaluate the quality of activities performed by all research organizations receiving public money, and by funding instruments focused on research and innovation [37]. Reports are included from the first and second evaluation rounds. VQR I covers the period 2004–2010, and included nine of the 14 disciplinary areas in the Italian system, while VQR II covers 2011–2014 with 11 disciplinary areas. Since each disciplinary committee has the mandate to determine the appropriate evaluation criteria within its field(s) of research, the reports of the different sectors were treated as individual bibliometric exercises in this meta-evaluation, although I found that bibliometric methods in VQR II were more streamlined than in VQR I. Thus, the VQR assessments were treated as 20 individual studies to my study sample (14% of study sample).
6. Finally, I searched the worldwide web for evaluation reports by funding agencies. Some countries and agencies follow high standards of transparency with regards to public research evaluation, including the Swedish Council for Science (VR), the Swedish Environmental Protection Agency (SEPA), the Danish Council for Strategic Research, and the British Wellcome Trust, among others. These web searches identified a total of 23 relevant and publicly available evaluation reports (17% of study sample).

The generalizability of the descriptive findings on professional practices hinges on the quality of the sampling process. Randomization was not possible due to the exploratory nature of the study. Relative to the size of their national research systems, some countries are strongly represented while others appear underrepresented. I am confident that this variation is to a large extent due to different levels of bibliometric activity, since some countries have a tradition of quantitative research assessment while others do not [26, 27, 32]. Relatedly, some professional actors have published a large share of the study sample while others have authored only one or two reports. Since my purpose is to describe the professional field, I have to deal with the fact that this field is dominated by certain actors holding a large “market share”. In order to deal with this unequal distribution and to compare different segments of professional practice, I decided to analyse the bibliometric methods by the five largest “dedicated” organizations (CWTS, NIFU, TR/ Clarivate, VQR, CSIC) separately from the rest of the “other” bibliometric experts.

The final study sample includes 138 distinct bibliometric studies, of which 102 (74%) evaluate research organizations and 36 (26%) evaluate funding instruments. The Italian VQR was the largest evaluation exercise within the sample in terms of number of researchers and publications under assessment. Since discretion on impact metrics was given to disciplinary committees, I treated the VQR as two rounds of parallel assessments to examine the methods used in each case (n = 20, S1 Table). The largest share of studies was produced by the CWTS, a contract research institute at Leiden University specializing in bibliometric assessment services (n = 37, S2 Table). NIFU is a non-university institute conducting research on the Norwegian and neighbouring Nordic science and innovation systems (n = 12, S3 Table). The assessment service by former Thomson Reuters Evidence Ltd, today part of the Web of Science Group owned by Clarivate Analytics, and abbreviated here as TR/ Clarivate, is represented with studies on funding instruments in the UK and EU (n = 7, S4 Table). The Spanish CSIC is an extra-university research organization that has its own internal evaluation unit (n = 5, S5 Table). Since studies from the same organization used identical citation impact metrics (CWTS, NIFU, TR/ Clarivate, CSIC), or at least shared important characteristics (VQR), I separately analysed the respective subsets with regards to some dimensions. The remaining 57 studies are labelled as studies by “other bibliometric experts” (S6 and S7 Tables for evaluations of research organizations and funding instrument). In this way, I can also observe if the methods by the “other” bibliometric experts are similar or different from those employed by prominent organizations, which is relevant for the question if the latter function as role models of methodological know-how. While the meta-evaluation was not designed as a country comparison, some national differences in assessment methodology are readily apparent as a result of this clustering.

Some uncertainty remains, since the coverage of individual nations does not only reflect the diffusion of bibliometric methods, but may also result from different national transparency policies. For example, evaluations of the German Max Planck Society are usually kept confidential. In contrast, publication is mandatory under transparency rules in Sweden, such that evaluation reports are generally available on the internet. In line with Abbott´s theory, the meta-evaluation approach should be considered in tandem with case studies of national jurisdictions, such as [32], to elucidate how science and higher education policies interact with the application and acceptance of bibliometric research assessment. There is no prima facie reason to assume that bibliometric techniques systematically differ between confidential sources and published reports, except with regards to the reported level of aggregation. For example, published reports by the Italian VQR and CWTS do not contain individual data although the reports state that the same methods were also applied at the level of individuals (VQR) or teams (CWTS). On the other hand, systematic methodological variation between countries with more transparency in contrast to countries with more confidential policies cannot be excluded. Consequently, the results of this study refer only to the available studies and cannot simply be generalized to unpublished work.

Analyses are presented cross-sectionally over the whole study period 2005–2019 in the main text. Longitudinal analyses comparing three five-year periods (2005–2009; 2010–2014; 2015–2019) are documented in supplementary materials (S8–S13 Tables). Bibliometric research assessment has expanded considerably during the period investigated from 2005–2019 and was fiercely debated among academics and evaluating agencies in several European countries. Also since the mid-2000s, academic publications on citation indicators soared, as documented in [19]. A comparison across time-periods mainly shows that bibliometric assessments spread to a larger number of countries within Europe, while an increasing influence of certain professional standards could not be asserted. In other words, while the present study covers fifteen years of expansion of bibliometric research assessment in Europe, institutional obstacles to bibliometric professionalism should be analysed in more detail as a next step [38].

Before answering the question if there are certain de-facto standards in terms of indicators used, I need to ask to what extent there is agreement in evaluation objectives. To a large extent, variation in study objectives is limited by the study selection criteria. The choice of citation indicators usually implies some sort of performance comparison between different units under study. It is important to understand that similar methods of performance assessment can be and are employed for widely different policy and management purposes [39]. For example, [21] argued that bibliometric evaluation was used by Dutch research organizations with the aim to identify promising young researchers that had not yet been fully established in terms of disciplinary reputation and networks, but also to put underperforming research areas on display in order to justify change action by university management. Quite differently, the Italian assessment exercise VQR was designed to inform central decision-making concerning the redistribution of national block funding between universities and academic departments. The important policy objectives behind the choice of metrics are not necessarily well documented in the bibliometric reports, and may even fluctuate during the policy process, since informal goals may be as influential as stated formal goals. Therefore, the meta-evaluation is confined to analysing formally stated goals, the frame of reference, and the main dimensions of comparison in each study for judging similarity in evaluation objectives.

Methodologically, this meta-evaluation is based on structured content analysis. I analysed the bibliometric design of each individual study using a scheme of 37 coding questions (S1 Appendix) in the following ten topical areas: (1) meta- information regarding the individual study, (2) the professional framework, (3) the object of evaluation, (4) the citation databases, (5) quality enhancement of the bibliometric raw data, (6) sampling strategy and data collection, (7) research fields under evaluation, (8) definition of citation data, (9) citation impact indicators, and (10) utilized statistical methods. Most items involved a nominal level of measurement, i.e. non-ordered qualitative characteristics. Five items were formulated as open questions, enabling raters to record more detailed information. This coding scheme was developed via an iterative procedure beginning with a partial sample. To test interrater reliability, two raters applied the initial coding scheme to an initial sample of 20 different studies. When coding differences became apparent, they were discussed among the two raters and the items were improved to reduce their ambiguity.

This remainder of this section comments on methodological choices involved in the design of the coding scheme (S1 Appendix). The topical area (2) “professional framework” was derived from sociological theory, distinguishing bibliometric experts working as external contractors from those who are employed as staff of the organization to be evaluated. Most other coding items were defined following the methodological literature on citation analysis, particularly [6, 8, 21].

Regarding (3) “the object of evaluation”, I distinguish research organizations and funding instruments, which pose different challenges for evaluation design. In general, publications are linked to authors and authors are linked to research organizations via their institutional affiliations. In this way, research organizations are treated as author aggregates, with large variations in scale. It is more difficult to attribute publications to individual funding instruments because authors, and author teams in particular, typically receive funding from diverse sources and there is a variable time lag between funding input and publication output. Only during the most recent years, funding acknowledgements were in a few cases applied for funding instruments evaluations, and are therefore not included in this analysis.

(4) “Citation databases” includes major multidisciplinary databases WoS and Scopus along with Google Scholar and other specialized disciplinary databases, such as PubMed for the medical sciences or MathSCInet for mathematics. These databases are commodities storing abstract knowledge according to [18].

(5) “Quality enhancement of bibliometric raw data” refers to the fact that quality of citation data as offered in conventional licences by WoS and Scopus is insufficient for most bibliometric purposes. It is at least necessary to disambiguate author names and institutional affiliations, but also journal names. Data quality also includes controls of the accordance between the actual research fields of an evaluation object and their operationalization for assessment purposes, which can be regarded as checks for external validity.

The topical areas (6) “sampling strategy and data collection”, (7) “research fields under evaluation”, (8) “definition of citation data” refer to methodical details of data collection, for example time period, treatment of self-citations, and citation windows of a given analysis.

(9) “Citation impact indicators” belongs to the core of the meta-evaluation. Since a large array of citation impact metrics have been proposed in the literature ([19] collected 169 indicator variants published 1972–2016), the question is how detailed the analysis should be in order to meaningfully describe convergence or divergence in terms of indicators. Based on widely used bibliometric reviews [6, 8, 40–42], I distinguish among six groups of metrics: (a) journal impact metrics, (b) field-normalized arithmetic mean, (c) other field-based percentiles, (d) h-index and h-type indices, (e) source-normalized metrics, (f) indirect impact metrics, and (g) other metrics as rest category, and to record combinations of these. An open question was provided in order to include the formula and methodological details of respective indicators. These six categories describe divergent measurement concepts on an aggregated level, but it is possible to examine variation within categories through the open question. The use of normalization was encoded separately including the underlying classification of scientific fields.

(10) “Utilized statistical methods” refers to information on the significance of performance differences across study units, but also includes an open item recording special features such as innovative tools for analysis or software.

## 4. Results

### 4.1 Bibliometric research assessment is most frequently used in the Nordic countries, followed by Italy, the Netherlands, and the United Kingdom

I found instances of bibliometric evaluation in many European countries, but the most regular use of bibliometric assessments during the observation period was concentrated in a few countries. Overall, my sample includes studies from 21 countries plus Framework Programs of the European Union. Approximately 26% of the studies, including four cross-country evaluations, were performed by the four Nordic countries Sweden, Norway, Finland, and Denmark, followed by Italy, the Netherlands, and the United Kingdom (Table 1). The Netherlands and the Nordic countries are medium-size research systems that show strong performance in international comparison. Among the larger public research systems in Europe, Italy is the only country that performs national-scale bibliometric assessment. The UK Research Excellence Framework only uses bibliometric data to inform peer review [9, 25], but the sample includes evaluations from several important British funding agencies, including the Medical Research Council and the Wellcome Trust. Germany has no national framework for research evaluation [43], and my search strategy did not yield any bibliometric assessments from France. A longitudinal analysis reveals that bibliometric assessment only recently spread to Eastern European countries, including Romania, Lithuania, Serbia, and Slovakia.

**Table 1:** Frequencies of studies across countries and evaluation objects.

Country	Acronym	Research organization	Funding instrument	Studies total*	% studies
Italy	IT	22	0	22	16
Netherlands	NL	17	1	18	13
United Kingdom	UK	8	8	16	12
Sweden	SE	8	6	14	10
Norway	NO	11	3	14	10
Germany	DE	8	3	11	8
Finland	FI	7	3	10	7
European Union (ERA)	EU	2	7	9	7
Spain	ES	7	1	8	6
Denmark	DK	4	3	7	5
Greece	GR	5	0	5	4
Austria	AT	2	2	4	3
Ireland	IE	1	2	3	2
Switzerland	CH	3	0	3	2
Hungary	HU	1	1	2	1
Romania	RO	2	0	2	1
Belgium	BE	1	0	1	1
Island	IS	1	0	1	1
Lithuania	LT	0	1	1	1
Luxemburg	LX	1	0	1	1
Serbia	RS	1	0	1	1
Slovakia	SK	1	0	1	1
Studies covering two or more countries		5	2	7	5
**Total counts**	** **	**114**	**41**	**154**	**100**
**Studies total**	** **	**102**	**36**	**138**	**––**

Meta-evaluation study set, 2005–2019

*Some studies cover evaluation objects from more than one country; thus, the sum of research organizations and funding instruments across countries is larger (n = 154) than the total number of studies (n = 138). The last column refers to the percentage of studies.

### 4.2 The Web of Science (WoS) is the dominant database for public research assessment in Europe

The bibliometric evaluation of public research in Europe during the observation period was largely based on the citation indices contained in the WoS. Of the total sample set, 87% of studies relied on WoS, while 21% used Scopus or a combination of WoS and Scopus (Table 2). The share of WoS decreased somewhat from 95% in 2005–2009 to 87% in 2015–2019, but the use of ‘WoS improved versions’ remained stable or increased slightly (S9 Table). The use of Scopus still derives in large part from the Italian VQR. Some studies employed designated databases, such as PubMed or MathSCInet, but these alternative citation databases exist for only a few disciplines. In other studies, citation data were complemented by national databases, which are more comprehensive in terms of research products but do not contain original citation data [44]. For example, the Norwegian Current Research Information System (CRISTIN) includes a larger array of document types, such as books and book chapters [27]; and the Italian VQR includes all types of research outputs, e.g. software, patents, maps, and artworks [37]. While 7% of studies relied on the search engine Google Scholar to cover a larger variety of sources, the TR/ Clarivate book citation index BKCI was not used at all within this sample [45].

**Table 2:** Databases used for bibliometric research assessment.

Databases	Studies total	% studies
Web of Science (WoS)	120	87
– WoS improved versions	66	48
Scopus	29	21
Google Scholar	10	7
Disciplinary databases (e.g. PubMed)	8	6
National research databases (e.g. Cristin)	33	24
Organization-specific databases	4	3
Patent database	1	1
**Studies total**	**138**	**100**

Meta-evaluation study set, 2005–2019

One important issue in bibliometric assessment is the extent to which bibliometric databases actually cover the investigated research fields [3, 21, 46]. Expert organizations have applied different methods to address this question of external validity. VQR and NIFU used their respective national publication databases to determine the coverage in international citation databases (external coverage) [47]. Using another approach, CWTS analysed the database coverage of the references cited within the studied publication sample (internal coverage) [21]. Among the studies by other bibliometric experts, only 18% investigated database coverage.

### 4.3 Expert organizations invest in the improvement of WoS citation data and set technical standards for data quality

Raw citation data, as provided by WoS or Scopus, require considerable processing before they are adequate for the assessment of authors and research organizations [48]. The main issues are the ambiguity of author names and institutional addresses, and the unambiguous assignment of authors to research institutions (Table 3). The correct disambiguation of institutional name variants requires detailed knowledge of national research systems. These and other technical problems can further lead to certain proportions of false citation linkages in the raw data [49]. Expert organizations—including CWTS, NIFU, the Italian Institute for System Analysis and Computer Science (IASI), the German Max Planck Society, and the German Competence Centre Bibliometrics—currently deal with this situation by buying raw data from database providers (Clarivate Analytics, formerly Thomson Reuters, sometimes complemented by Scopus Elsevier) and constructing in-house databases with improved data quality. Access to WoS citation data of improved quality was available to 48% of the total study sample, including studies directly by TR/ Clarivate, but only to 19% of studies by other bibliometric experts. Consequently, studies by the other bibliometric experts also frequently mention the effort required for disambiguation of institutional addresses (51%) and author names (47%). Not surprisingly, one of the main practical arguments for the h-index is its greater robustness with regards to incomplete publication and citation data.

**Table 3:** Enhanced data quality.

Improvement	Dedicated orgs.	Other experts	Studies total	% dedicated orgs	% other experts	% studies total
WoS improved versions	55	11	66	68	19	48
Institutional adresses cleaned	75	29	104	93	51	75
Author names disambiguated	64	27	91	79	47	66
Corrections for self-citations	37	15	52	46	26	38
Database coverage*	52	10	62	64	18	45
Validity of field definition**	6	7	13	7	12	9
Check of publication lists by authors	8	12	20	10	21	14
**Studies total**	**81**	**57**	**138**	**100**	**100**	**100**

Meta-evaluation study set, 2005–2019

* ‘Database coverage’ refers to analyses of internal or external coverage of scientific fields by citation databases.

** ‘Validity of field definition’ refers to the congruence between a bibliometric field definition and the targeted field of research. This is seldom checked empirically.

A related issue is the need to verify the completeness of publication records. If a completed analysis were later found to be missing individual highly cited papers, this could seriously jeopardize assessment credibility in the eyes of stakeholders. Italy and Norway impose a mandatory requirement for each scientist to register a certain number of publications (Italy) or all publications (Norway). These national publication records provide the basis for author searches of citation databases for evaluation purposes. CWTS uses a different approach, collecting internal publication records from research organizations, and sometimes sending these records to the authors for personal verification and completion. Personal verification by authors was also used by 21% of the studies by other bibliometric experts.

### 4.4 Citation impact is most frequently assessed with reference to international scientific fields

As stated in the method section, the formal evaluation objectives are broadly homogeneous in that they all involve a comparative assessment of research performance. This section further examines the frame of reference, i.e. how the relevant comparison for performance assessment was construed in each case. It is striking that the analysed publication samples vary in size by orders of magnitude. For research organizations, the modal size category is 1,000–10,000 publications. In contrast, for funding instruments, the modal category is 10,000–100,000 publications. There are a total of eight studies with sample sizes over 100,000 publications, five of which are in the area of medical sciences, and three investigate multiple research organizations. (Table 4).

**Table 4:** Sample sizes in bibliometric evaluation studies.

Sample size*	Research organization	Funding instrument	total	% research org.	% funding inst.	% total
100–1,000	13	7	20	13	19	14
1,001–10,000	51	7	58	50	19	42
10,001–100,000	17	15	32	17	42	23
>100,000	7	1	8	7	3	6
Missing data	14	6	20	14	17	14
**Studies total**	**102**	**36**	**138**	**100**	**100**	**100**

Meta-evaluation study set, 2005–2019

*Total number of publications that an assessment is based upon.

To analyse the frame of reference in greater detail, research organizations were differentiated according to scale (number of institutes or universities) and scope (mono-disciplinary vs. multi-disciplinary), while funding instruments were distinguished according to type of unit funded (research projects, scientists, research organizations, or portfolio review) (rows in Table 5). Concerning performance measurement, I distinguished between those with “international field comparison”, “national rankings”, and “other” (columns in Table 5). International field comparison refers to the assessment of observed citation rates with reference to the expected citation rates for the same research field and time period (often also for the same document type) throughout the entire database [6, 50]. This type of measurement was used in 64% of the study sample, including 55 studies by CWTS, NIFU, and TR/ Clarivate (except B6). In contrast, with national rankings, the relative national position defines research performance. This type of measurement was used in 25% of studies, including all 20 studies by the VQR I-II [37], but also 14 studies by other bibliometric experts. While international field comparisons occurred across all categories of evaluation objects (rows in Table 5), national rankings were used only for the comparison of departments and institutes, often by h-index/ h-type indices within single fields (rows 1.3; 1.4). The remaining category “other” contains mainly inter-group comparisons, including some quasi-experimental designs (funded vs. non-funded scientists) in studies on funding instruments. Dedicated organizations used international field comparisons more frequently (70%) than other bibliometric experts (56%). Notably, I identified the divergence of the Italian VQR from the dominant approach in professional evaluation practice.

**Table 5:** Frame of reference for research assessment.

	Evaluation object (unit of analysis)	Internat. field comp.	National ranking	Other	Studies total	% studies
1.1	*Single* research organization in a *single* field	19	––	2	**21**	**15**
1.2	*Single* research organization with *multiple* fields (i.e. univ. with several departments)	17	––	0	**17**	**12**
1.3	*Several* research organizations in a *single* field (i.e. departments, institutes)	14	11	2	**27**	**20**
1.4	*Several* research orgs. with *multiple* fields (i.e. univ. with departments, non-univ. inst.)	5	23	0	**28**	**20**
1.5	Umbrella research orgs. with *several* institutes (i.e. non-university research institutes)	3	0	6	**9**	**7**
	**Research organizations total**	**58**	**34**	**10**	**102**	**74**
2.1	Projects funded	7	––	2	**9**	**7**
2.2	Scientists funded	6	0	3	**9**	**7**
2.3	Research organizations funded	8	0	0	**8**	**6**
2.4	Evaluation of funding portfolio	10	0	0	**10**	**7**
	**Funding instruments total**	**31**	**0**	**5**	**36**	**26**
	**Studies total**	**89**	**34**	**14**	**138**	**100**
	**% Studies**	**64**	**25**	**10**	**100**	**––**

Meta-evaluation study set, 2005–2019

Rows distinguish evaluation objects while columns distinguish performance standards. Both dimensions contribute to the frame of reference in evaluation studies. This synopsis is based on the current sample and does not display all possible combinations.

The predominance of international field comparisons is affected by three dedicated organizations: CWTS, NIFU, and TR/ Clarivate. A closer look at the subset of 32 studies by other bibliometric experts with international field comparisons reveals two important sources of influence on their choice of methods: at least ten studies explicitly use indicators by and refer to authors from CWTS in their method section (F1; F6; F7; F8; F20; F27; F29; G5; G8c; G12), documenting the status of CWTS as a leading expert organization. Second, in eight cases, expected citation rates were purchased directly from Thomson Reuters (F12), based on Essential Science Indicators (F3, G15) or Incites (F19; F20; F27; G18; G19), in addition to the seven bibliometric assessments commissioned directly from TR/ Clarivate. Fewer studies used Scopus (F16; F18; F26; F28; F30) or Google Scholar (F18; G5; G14) as alternative or additional sources. These findings show that while there remains considerable variation in the details of the calculation of international field averages during the period observed, a leading expert organization and a single database provider are important sources of conceptual and methodological convergence.

### 4.5 The WoS classification of science fields functions as a de facto reference standard for research performance assessment

Dedicated organizations generally used field-normalized indicators for impact assessment, most frequently either field-normalized top-percentiles, field-normalized arithmetic mean, or both (Table 6), while a more diverse picture emerges for other bibliometric experts. Some individual experts adhered to the same professional framework as defined by CWTS, NIFU, and TR/Clarivate, based on international field comparison and field-normalization (39%). Examples include bibliometricians at the Swedish Science Council, the German Max-Planck Society, or studies by the Canadian expert organization Science Metrix. But there are also experts more remote from the core of academic bibliometrics, who tend to choose different methods. First, there are authors from other disciplines, mostly medicine, but also economics, conducting bibliometric assessments within their own specialties (medicine: F1; F11; F12; F24; F31; F37; F38; economics: F14; F33). Second, several studies are from countries that were for a long time peripheral to the European science system, and where research evaluation started only recently, including Greece (F13; F21; F22; F23; F36), Romania (F25a,b; F34), Lithuania (G18), and Slovakia (F33). Third, there are bibliometric studies questioning the cost-effectiveness and usefulness of comprehensive peer-review based evaluation schemes in Italy, United Kingdom, and Romania (F4a,b; F25a,b; F26a,b; F34). These three groups of studies have in common that they used other bibliometric indicators than the dedicated organizations, in particular CWTS, NIFU, and TR/ Clarivate. They often employ the h-index (sometimes other h-type indices) (46%), journal impact factor (46%), and often do not have access to citation data of highest quality. By contrast, dedicated organizations only rarely used h-type indices (4%) and more often use impact-level categories of journals (16%) for scientific fields with insufficient coverage in citation databases (Table 6, 2.4).

**Table 6:** Types of impact metrics used in evaluation studies.

	Type of metric	Dedica-ted orgs.	Other experts	Studies total	% dedica-ted orgs.	% other experts	% studies total
**1.**	**Observed citation impact**	**80**	**46**	**126**	**99**	**81**	**91**
1.1.	Field-normalized impact total	78	22	100	95	39	72
1.1.1.	Field-normalized arithmetic mean (e.g. MNCS)	57	19	76	70	33	55
1.1.2	Field-normalized percentiles (e.g. 10% top-cited publications)	61	17	78	75	30	57
1.2	H-index and h-type indicators	3	26	28	4	46	21
1.3	Other observed impact	70	23	93	86	40	67
**2.**	**Journal impact**	**38**	**29**	**67**	**47**	**51**	**49**
2.1	Journal impact factor (e.g. 2-year, 5-year);	28	26	54	35	46	39
2.2	Indirect journal impact (e.g. Eigenfactor metrics)	14	3	17	17	5	12
2.3	Source normalized journal impact (e.g. SNIP)	0	2	2	0	4	1
2.4	Impact-level categories for journals (e.g. Norwegian model)	13	3	16	16	5	12
	**Studies total**	**81**	**57**	**138**	**100**	**100**	**100**

Meta-evaluation study set, 2005–2019

In principle, field normalization is applicable to different types of citation metrics [6], including arithmetic mean, highly cited percentiles, h-type indices, and indirect citation metrics, and also journal impact. To avoid issues regarding field normalization, exclusively source-normalized impact metrics have been construed as a methodological alternative [41]. However, only some of the possible combinations are actually found in my sample. Eight studies used JIF-Quartiles, a variant of field-normalized journal impact (included in row 2.2), but no study uses a field-normalized h-index. No study calculated either indirect citation (prestige) indicators or source normalized indicators for observed citations. Instead, citation databases provided indirect and source normalized journal impact metrics including Eigenfactor metrics (TR/ Clarivate) and SNIP (Scopus) in recent years. In general, the overview in Table 6 reveals that research assessment practice during the study period included few of the methodological inventions that have recently been proposed in the academic debate on impact metrics [8, 19, 51].

Journal impact was used quite frequently (49%), despite repeated criticism that the substitution of a journal’s impact for the actual number of citations lacks validity [52, 53]. While 91% of studies report observed citation data, nevertheless journal impact was often used to substitute missing data, either because publications are so recent that actual citations are not yet available (e.g. VQR), or because articles are published in journals that are not covered by the database (e.g. NIFU and VQR). Notably, journal impact metrics are easily accessible via Journal Citation Reports, which is especially relevant for bibliometricians lacking fully licensed access to citation databases. Sometimes journal impact was used very pragmatically, for example simply for distinguishing between two broad levels of journal quality (Table 6, 2.4).

Overall, 78% of all studies used field normalization, including the field-normalized arithmetic mean, other field-related percentiles, and field-normalized journal impact (Table 7). Among these 107 studies, 83% relied on the WoS classification of science fields (WoS subject categories SCs), and an additional 5% used the related Essential Science Indicators classification by TR/ Clarivate. The Scopus science classification was mainly used in the Italian VQR. Of all field normalizations, 17% were based on self-defined journal sets, sometimes combined with keywords. Both, the alternative journal-based classifications proposed in the academic literature, and the publication-based clusters by CWTS, have been developed to overcome methodological problems associated with the WoS classification of science fields. CWTS actually replaced the WoS classification by a taxonomy of approximately 4000 clusters since about 2016 [54]. Yet while alternative journal-based taxonomies have had little influence to date, and have not been used at all by dedicated organisations, the publication-based clusters, on the other hand, are proprietary and thus not available for use by other professionals. It can be concluded that thus far the WoS classification of science fields has retained the status of a de facto reference standard.

**Table 7:** Classification of science fields used for field normalization.

Classification	Dedicated orgs.	Other experts	Studies total	% dedicated orgs.	% other experts	% studies total
Web of Science Classification	66	23	89	84	40	64
Scopus Classification	16	3	19	20	5	14
Essential Science Indicators	2	3	5	2	5	4
Alternative journal-based classification*	0	5	5	0	9	4
Self-defined journal sets	9	4	13	11	7	9
Keywords combined with journal sets	3	2	5	4	4	4
Publication-based clusters	7	0	7	7	0	5
Other	2	4	6	2	7	4
Studies with field normalization	79	28	107	98	49	78
**Studies total**	**81**	**57**	**138**	**100**	**100**	**100**

Meta-evaluation study set, 2005–2019

* This category includes classifications proposed in bibliometric literature, i.e. [55–57]; OST classification.

There has been some debate in the literature regarding the adequacy of WoS SCs as the basis for field normalization. One point of concern is the lack of transparency regarding the methodology used to construct and update the categories [56, 58]. One study investigated the adequacy of the WoS classification, and empirically demonstrated that very few journals in the WoS were miscategorized in terms of their direct citation relations [58]. These authors found that some journals in the WoS do not display strong citation relations with their present category, but these journals generally appeared to be located between fields rather than to have been suboptimally assigned. Overall, WoS performs significantly better than Scopus with regards to the adequacy of journal categorization, which is related to the fact that journals belong to fewer categories in WoS than in Scopus.

Notably, [58] do not address the more fundamental question of how journal citation clusters are distributed across WoS SCs. Random walk models demonstrate that the total inter-journal citation network is characterized by densely connected regions and areas of much lower citation traffic [59]. The WoS SCs substantially vary in size; thus, it seems likely that some categories contain several clusters while others may comprise only one or even no cluster at all. [60] demonstrated that the WoS category “Library and information sciences” includes two clearly distinguishable journal citation clusters, and that eight journals publishing research in “Science and technology studies” belong to eleven different WoS SCs. Concerns have been raised that the internal heterogeneity of SCs with regards to research topics and citation densities may pose serious problems for field normalization [61]. One possible way to address this problem are more fine-grained publication-based clusters [54].

## 5. Discussion

In this paper, I analysed the methods used in 138 bibliometric evaluation studies, from a meta-perspective informed by Abbott’s theory of professions [17, 18]. In contrast to conventional meta-evaluations that assess how well a set of evaluation studies adheres to predefined methodological standards, the purpose of my meta-evaluation was to investigate whether professional de facto standards could be observed in bibliometric research assessment. More precisely, this study posed two research questions. First, what were the prevailing methods of bibliometric performance assessment in European evaluation practice during the period 2005–2019? Second, if methodological de facto standards existed, which actors were in the position to define them?

A detailed review of assessment methods revealed that bibliometric assessment was more frequently performed in the Nordic countries, Italy, the Netherlands, and the United Kingdom, and that WoS was the dominant database used for public research assessment across 21 European countries. Expert organizations that invest in improving WoS citation data were able to set technical standards with regards to data quality. Citation impact was most frequently assessed with reference to the WoS classification of science fields (SCs), which thus far retained the function of a de facto reference standard.

My findings demonstrate two main choices regarding the design of bibliometric research assessments. First, there is the choice between international field comparison, national ranking, and other designs, such as inter-group comparisons, as the main standard of performance. Expert organizations such as CWTS, NIFU, and TR/ Clarivate clearly define international field comparison as the predominant professional framework across all categories of evaluation objects, while the Italian VQR deviates from this professional standard by assessing Italian universities via national rankings on composite performance indicators that are unknown elsewhere. Italy does not follow a model of bibliometric professionalism but applies bibliometric assessment as an element of central state governance of universities [38].

The second and related choice concerns field-normalized citation impact versus h-index and h-type indices. I found that all dedicated organizations use field-normalized citation impact, while h-type indices are more prevalent among those bibliometric experts that do not have access to high-quality citation data, either because the authors come from disciplines applying bibliometric assessment, often medicine, or from countries located at the periphery of the European science system.

These findings provide support to [32] that highlighted to key role of expert organizations in shaping both the academic and professional field of bibliometric evaluation. The analysed study set clearly documented the prominent position of expert organizations in the field. The two most prominent organizations were CWTS and NIFU, both of which have regularly conducted bibliometric assessments for many years, and have produced important shares of the data set. These expert organizations were able to define technical standards with regards to enhanced quality of publication and citation data. Following the example of CWTS, NIFU and other expert organizations (e.g. the Italian Institute for System Analysis and Computer Science, the German Max Planck Society, and the German Competence Centre Bibliometrics) have invested in establishing in-house databases to clean WoS raw data. Bibliometric experts lacking equivalent databases cannot attain the same level of data quality, at least not for large publication quantities.

Perhaps more important than identifying the leading roles of CWTS and NIFU as bibliometric expert organizations, my analysis unequivocally documents the predominance of TR/ Clarivate in terms of defining methodological standards for the performance assessment of public research in Europe. During the observation period, the provider of WoS assumed the most important role in defining de facto standards for bibliometric assessment. All expert organizations, including CWTS and NIFU, based their citation analyses on data licensed by Clarivate Analytics/Thomson Reuters, as did most other bibliometric experts. However, Clarivate Analytics (via its licencing policy) regulates the extent to which different user groups can access citation data. Moreover, WoS subject categories retained their function as de facto reference standards for bibliometric assessment. In addition, there is the effective dissemination of selected impact indicators via the Journal Citation Reports and Incites. Although academic bibliometricians have endeavoured to develop alternative categorizations of scientific fields [56, 62, 63] or more complex impact indicators, such efforts have had little impact on professional practice so far because they are not distributed alongside with citation data. I found a few examples of the use of alternative or supplementary sources, such as the specialized citation database Medline/Pubmed and the Norwegian documentation system CRISTIN. These examples further underline that bibliometric evaluation practice depends first and foremost on the data sources that are accessible for comparative analyses of research performance.

These findings suggest that the current ownership structure of the most widely used citation databases has had a restraining effect on the development and diffusion of professional bibliometric methods during the observation period. First, in the current situation, the development of new diagnostic techniques within the academic sector has remained largely disconnected from their application and diffusion in the professional field. A case in point is the development of alternatives for the WoS classification of science fields. A likely explanation for why alternative academic journal classifications have not spread further consists in that they can only be implemented with appropriate inhouse-databases. In contrast, WoS or Scopus science categories are distributed by database providers alongside with WoS / Scopus raw citation data. The CWTS publication-based clusters, on the other hand, represent a professional solution, but because this solution is proprietary, it cannot be widely shared, discussed, and improved in the academic sector.

Second, I find that bibliometric methods have spread to a larger number of countries in Europe over the observation period. In principle, the scientific development in countries seeking to catch up scientifically as well as economically should not be assessed with second-rate methods. Lack of access to first-rate citation databases seems to have influenced the methodological choices of bibliometric assessments in countries such as Greece, Romania, Lithuania, and Slovakia. From these instances I infer that shared access to citation databases would be an important mechanism to better connect methodological developments from the academic sector with their application and diffusion in professional assessment practice.

The theoretical framework used in this study raises questions regarding professional control over the production of and access to high-quality citation data and analysis tools. Abbott asked how corporate control of expert commodities (in this setting, citation databases) will affect the future development of professional knowledge and practice [18]. It seems likely that an open-access regime of citation databases would support the development and broad diffusion of more sophisticated bibliometric techniques for research assessment. Therefore, it seems promising to explore more explicit connections between methodological debates in evaluative bibliometrics and the ongoing open access transformation of the scientific publication system [64–66]. Further research and policy discussions should focus on whether and how open access to citation data could be provided in Europe.

## Supporting information

S1 Table Evaluation studies by the Italian Valutazione della Qualità della Ricerca VQR 2004–2010, published in 2013. Evaluation studies by the Italian Valutazione della Qualità della Ricerca VQR 2011–2014, published in 2017.
(DOCX)

S2 Table Evaluation studies by the Centre for Science and Technology Studies CWTS, Leiden 2005–2019. (DOCX)

S3 Table Evaluation studies by the Nordic Institute for Studies in Innovation, Research and Education NIFU, Oslo, 2005–2019. (DOCX)

S4 Table Evaluation studies with bibliometric analysis by Thomson Reuters Evidence Ltd/ Clarivate Analytics, London, 2005–2019. (DOCX)

S5 Table Evaluation studies by the Spanish Council for Scientific Research CSIC, 2005–2019. (DOCX)

S6 Table Evaluation studies of research organizations by other bibliometric experts 2005–2019. (DOCX)

S7 Table Evaluation studies of funding instruments by other bibliometric experts 2005–2019. (DOCX)

S8 Table Frequency of studies across countries and time periods. (DOCX)

S9 Table Databases for bibliometric assessment and time periods. (DOCX)

S10 Table Enhanced data quality and time periods. (DOCX)

S11 Table Frame of reference for research assessment and time periods. (DOCX)

S12 Table Type of impact metrics and time periods. (DOCX)

S13 Table Classification of science for field normalization and time periods. (DOCX)

S1 Appendix Meta-evaluation coding questions. (DOCX)

## References

[1] Cagan R. The San Francisco Declaration on Research Assessment. Disease Models & Mechanisms. 2013;6:Editorial. 10.1242/dmm.012955 PMC3701204 23690539

[2] Adler R, Ewing J, Taylor P. Citation Statistics: A Report from the International Mathematical Union (IMU) in Cooperation with the International Council of Industrial and Applied Mathematics (ICIAM) and the Institute of Mathematical Statistics (IMS). Statistical Science. 2009;24(1):1–14.

[3] Bonaccorsi A, editor. The evaluation of research in social sciences and humanities Lessons from the Italian experience: Springer International Publishing; 2018.

[4] de Rijcke S, Wouters PF, Rushforth AD, Franssen TP, Hammarfeldt B. Evaluation practices and effects of indicator use. A literature review. Research Evaluation. 2016;25(2):161–9.

[5] Barré R. Les indicateurs sont morts, vive les indicateurs! Towards a political economy of S&T indicators: A critical overview of the past 35 years. Research Evaluation. 2019;28(1):2–6.

[6] Waltman L. A review of the literature on citation impact indicators. Journal of Informetrics. 2016;10:365–91.

[7] Mingers J, Leydesdorff L. A review of theory and practice in scientometrics. European Journal of Operational Research. 2015;246:1–19.

[8] Todeschini R, Baccini A. Handbook of bibliometric indicators: quantitative tools for studying and evaluating research: Wiley VCH, Weinheim, Germany; 2016.

[9] Wilsdon J, Allen L, Belfiore E, Campbell P, Curry S, Hill S, et al The Metric Tide: Report of the Independent Review of the Role of Metrics in Research Assessment and Management. HEFCE: HEFCE, 2015.

[10] EC. Assessing Europe´s University-based Research. Expert Group on Assessment of University-based Research. Brussels: European Commission, Directorate-General for Research RTD.C4, 2010 EUR 24187 EN.

[11] Braithwaite J, J H, K C, et al Comprehensive Researcher Achievement Model (CRAM): a framework for measuring researcher achievement, impact and influence derived from a systematic literature review of metrics and models. BMJ Open. 2019;9:e025320 10.1136/bmjopen-2018-025320 30928941 PMC6475357

[12] Cooksy LJ, Caracelli VJ. Quality, Context, and Use. Issues in Achieving the Goals of Metaevaluation. American Journal of Evaluation. 2005;26(1):31–42.

[13] Lam S, Dodd W, Whynot J, Skinner K. How is gender being addressed in the international development evaluation literature? A meta-evaluation. Research Evaluation. 2019;0(0):1–11.

[14] Stufflebeam DL. The Metaevaluation Imperative. American Journal of Evaluation. 2001;22(2):183–209.

[15] Good B. Assessing the effects of a collaborative research funding scheme: An approach combining meta-evaluation and evaluation synthesis. Research Evaluation. 2012;21:381–91.

[16] Waltman L, van Eck NJ, Visser MS, Wouters P. The elephant in the room: The problem of quantifying productivity in evaluative scientometrics. Journal of Informetrics. 2016;10:671–4.

[17] Abbott A. The system of professions: An essay on the division of expert labor. Chicago: University of Chicago Press; 1988.

[18] Abbott A. The Future of Professions: Occupation and Expertise in the Age of Organisation. Research in the Sociology of Organisations. 1991;8:17–42.

[19] Jappe A, Pithan D, Heinze T. Does bibliometric research confer legitimacy to research assessment practice? A sociological study of reputational control, 1972–2016. PLoS One. 2018;13(6):e0199031 10.1371/journal.pone.0199031 29902239 PMC6002049

[20] Miller PP, M. Accounting, organizing and economizing: connecting accounting research and organization theory. The Academy of Management Annals. 2013;7(1):557–605.

[21] Moed HF. Citation Analysis in Research Evaluation. Dordrecht: Springer; 2005.

[22] Power M. The Audit Society: Rituals of Verification. Oxford: Oxford University Press; 1997.

[23] Strathern M. Audit Cultures: Anthropological Studies in Accountability, Ethics and the Academy. London: Routledge; 1996.

[24] Ancaiani A, Anfossi AF, Barbara A, Benedetto S, Blasi B, et al Evaluating scientific research in Italy: The 2004–10 research evaluation exercise. Research Evaluation. 2015:242–55.

[25] Geuna A, Piolatto M. Research assessment in the UK and Italy: Costly and difficult,but probably worth it (at least for a while). Research Policy. 2016;45:260–71.

[26] Hicks D. Performance-based university research funding systems. Research Policy. 2012;41(2):251–61.

[27] Sivertsen G. Publication-Based Funding: The Norwegian Model 2016 In: Research Assessment in the Humanities: Towards Criteria and Procedures [Internet]. Zürich: Springer Open; [79–90].

[28] Sivertsen G. Unique but still best practice? The Research Excellence Framework from an International Perspective. Palgrave Communications. 2017;3:17078.

[29] Sivertsen G. Data integration in Scandinavia. Scientometrics. 2016;106:849–55.

[30] http://www.anvur.it/attivita/vqr/ [28 Jan 2020].

[31] https://www.risis2.eu/ [26 Mar 2020]

[32] Petersohn S, Heinze T. Professionalization of bibliometric research assessment. Insights from the history of the Leiden Centre for Science and Technology Studies (CWTS). Science and Public Policy. 2018;(45):565–78.

[33] https://ec.europa.eu/info/research-and-innovation/strategy/era_en [21 Feb 2019].

[34] Martin BR. The use of multiple indicators in the assessment of basic research. Scientometrics. 1996;36(3):343–62.

[35] Moed H, Halevi G. Multidimensional Assessment of Scholarly Research Impact. Journal of the Association for Information Science and Technology. 2015;66(10):1988–2002.

[36] http://si-per.eu/Home/About [28 Jan 2020].

[37] ANVUR. Valutazione della Qualità della Ricerca 2004–2010 (VQR 2004–2010) Rapporto finale ANVUR Parte Prima: Statistiche e risultati di compendio. Agenzia Nazionale di Valutazione del sistema Universitario e della Ricerca ANVUR: Agenzia Nazionale di Valutazione del sistema Universitario e della Ricerca ANVUR, 2013.

[38] Heinze T, Jappe A. Quantitative science studies should be framed with middle-range theories and concepts from the social sciences. Quantitative Studies of Science. 2020;1:1–17.

[39] Milzow K, Reinhardt A, Söderberg S, Zinöcker K. Understanding the use and usability of research evaluation studies. Research Evaluation. 2019;28(1):94–107.

[40] Egghe L. The Hirsch Index and Related Impact Measures. Annual Review of Information Science and Technology. 2010;44:65–110.

[41] Waltman L, van Eck NJ. Source normalized indicators of citation impact: an overview of different approaches and an empirical comparison. Scientometrics. 2013;96:699–716.

[42] Fragkiadaki E, Evangelidis G. Review of the indirect citations paradigm: theory and practice of the assessment of papers, authors and journals. Scientometrics. 2014;99:261–88.

[43] Biesenbender S. The governance and standardisation of research information in different science systems: A comparative analysis of Germany and Italy. Higher Education Quarterly. 2019;73:116–27.

[44] Sīle L, Pölonen J, Sivertsen G, Guns R, Engels T, et al Comprehensiveness of national bibliographic databases for social sciences and humanities: Findings from a European survey. Research Evaluation. 2018;27(4):310–22.

[45] Gingras Y, Khelfaoui M. Do we need a book citation index for research evaluation? Research Evaluation. 2019;28:383–93.

[46] Larsen PO, von Ins M. The rate of growth in scientific publication and the decline in coverage provided by Science Citation Index. Scientometrics. 2010;84:575–603. 10.1007/s11192-010-0202-z 20700371 PMC2909426

[47] Sivertsen G. Publication-Based Funding: The Norwegian Model. Zürich: Springer Open; 2016.

[48] Van den Besselaar P, Sandström U. What is the Required Level of Data Cleaning? A Research Evaluation Case. Journal of Scientometric Research. 2016;5(1):7–12.

[49] Franceschini F, Maisano D, Mastrociacomo L. Research quality evaluation: comparing citation counts considering bibliometric database errors. Quality & Quantity. 2015;49:155–65.

[50] Moed HF, de Bruin RE, van Leeuwen TN. New Bibliometric Tools for the Assessment of National Research Performance—Database Description, Overview of Indicators and First Applications. Scientometrics. 1995;33(3):381–422.

[51] Wildgaard L, Schneider JW, Larsen B. A review of the characteristics of 108 author-level bibliometric indicators. Scientometrics. 2014;101:125–58.

[52] Abramo G, D´Angelo CA. Refrain from adopting the combination of citation and journal metrics to grade publications, as used in the Italian national research assessment exercise (VQR 2011–2014). Scientometrics. 2016;109:2053–65.

[53] Hicks D, Wouters P, Waltman L, de Rijke S, Rafols I. The Leiden manifesto for research metrics. Nature. 2015;520:429–31. 10.1038/520429a 25903611

[54] Waltman L, van Eck NJ. A New Methodology for Constructing a Publication-Level Classification System of Science. Journal of the American Society for Information Science and Technology. 2012;63(12):2378–92.

[55] Glänzel W, Schubert A. A new classification scheme of science fields and subfields designed for scientometric evaluation purposes. Scientometrics. 2003;56(3):357–67.

[56] Archambault E, Beauchesne OH, Caruso J. Towards a Multilingual, Comprehensive and Open Scientific Journal Ontology. Proceedings of the 13th International Conference of the International Society for Scientometrics and Informetrics. 2011:66–77.

[57] Sandström U, Sandström E. Resurser för citeringar. 2008.

[58] Wang Q, Waltman L. Large-scale analysis of the accuracy of the journal classification systems of Web of Science and Scopus. Journal of Informetrics. 2016;10:347–64.

[59] Rosvall M, Bergstrom CT. Multilevel Compression of Random Walks on Networks Reveals Hierarchical Organization in Large Integrated Systems. PLOS One. 2011;6(4):e18209 10.1371/journal.pone.0018209 21494658 PMC3072965

[60] Leydesdorff L, Bornmann L. The Operationalization of "Fields" as WoS Subject Categories (WCs) in Evaluative Bibliometrics: The Cases of "Library and Information Science" and "Science & Technology Studies". Journal of the Association for Information Science and Technology. 2016;67(3):707–14.

[61] van Eck NJ, Waltman L, Van Raan AFJ, Klautz RJM, Peul WC. Citation Analysis May Severely Underestimate the Impact of Clinical Research as Compared to Basic Research. PLoS ONE. 2013;8(4):e62395 10.1371/journal.pone.0062395 23638064 PMC3634776

[62] Shu F, Julien C-A, Zhang L, Qiua J, Zhang J, Lariviere V. Comparing journal and paper level classifications of science. Journal of Informetrics. 2019;13:202–25.

[63] Ruiz-Castillo J, Waltman L. Field-normalized citation impact indicators using algorithmically constructed classification systems of science. Journal of Informetrics. 2015;9:102–17.

[64] Jubb M. Peer review: The current landscape and future trends. Learned Publishing. 2016;29:13–21.

[65] Piwowar H, Priem J, Larivière V, Alperin JP, Matthias L, Norlander B, et al The state of OA: a large-scale analysis of the prevalence and impact of Open Access articles. PeerJ. 2018;6:e4375 10.7717/peerj.4375 29456894 PMC5815332

[66] Wang X, Cui Y, Xu S, al. e. The state and evolution of Gold open access: a country and discipline level analysis. ASLIB Journal of information management. 2018;70(5):573–84.

