# Supplementary material for: Professional standards in bibliometric research evaluation? A meta-evaluation of European assessment practice 2005–2019
Source: PLoS One. 2020 Apr 20;15(4):e0231735. doi: 10.1371/journal.pone.0231735 (PMC7170233; doi:10.1371/journal.pone.0231735)
Supplement: S1 Table — Evaluation studies by the Italian Valutazione della Qualità della Ricerca VQR 2011–2014, published in 2017. (DOCX) [file pone.0231735.s001.docx]

**S1a Table. Evaluation studies by the Italian Valutazione della Qualità della Ricerca VQR 2004-2010, published in 2013.**

| **ID** | **Evaluation object** | **EO** | **Research Field** | **Authors*** | **Titel of VQR ANVUR Report with Annexe** |
| --- | --- | --- | --- | --- | --- |
| A1a | Universites and non-university research in 10 disciplinary subfields | RO | Mathematics and Informatics | Quarteroni AM, Abate M, et al. | Rapporto finale di area Gruppo di Esperti della Valutazione dell’Area 01 – Scienze Matematiche e Informatiche (GEV01) |
| A2a | Universites and non-university research in 8 disciplinary subfields | RO | Physical Sciences | Parisi G, Bracco A, Guerra F, Mattarese S, Ferdeghini C, et al. | Rapporto finale di Area Gruppo di Esperti della Valutazione dell’Area 02 (GEV02) – Scienze Fisiche |
| A3a | Universites and non-university research in 12 disciplinary subfields | RO | Chemistry | Barone V, Torsi L, Pacchioni G, Prato M, et al. | Rapporto finale di area Gruppo di Esperti della Valutazione dell’Area 3 (GEV03) – Chimica |
| A4a | Universites and non-university research in 12 disciplinary subfields | RO | Geosciences | Giorgi F, et al. | Rapporto finale di area Gruppo di Esperti della Valutazione dell’Area 04 (GEV04) – Scienze della Terra |
| A5a | Universites and non-university research in 21 disciplinary subfields | RO | Biological Sciences | Boero F, Rezzani R, Degli Esposti M, Geppetti P, et al. | Rapporto finale di Area Gruppo di Esperti della Valutazione dell’Area 05 (GEV05) – Scienze Biologiche |
| A6a | Universites and non-university research in 50 disciplinary subfields | RO | Medicine | Sesti G, Pozzan T, Paolisso G, Bellantone RD, Mutti A, et al. | Rapporto finale di Area Gruppo di Esperti della Valutazione dell’Area 06 (GEV06) – Scienze Mediche |
| A7a | Universites and non-university research in 30 disciplinary subfields | RO | Agrarian and Veterinary Sciences | Morelli L, Varanini Z, Scanziani E, et al. | Rapporto finale di area Gruppo di Esperti della Valutazione dell’Area 07 (GEV07) – Scienze agrarie e veterinarie |
| A8a | Universites and non-university research in 22 disciplinary subfields | RO | Engineering and Architecture | Garofalo F, Auricchio F, Olmo C, et al. | Rapporto finale di area Gruppo di Esperti della Valutazione dell’Area Ingegneria e Architettura (GEV08) |
| A9a | Universites and non-university research in 42 disciplinary subfields | RO | Industrial and Information Engineering | Ajmone MM, Garetti M, Sangiorgio E, Tognotti L, et al. | Rapporto finale di area Gruppo di Esperti della Valutazione dell’Area 09 (GEV09) – Ingegneria Industriale e dell’Informazione |

* Refers to the president and coordinators et al. of the expert groups leading the respective disciplinary evaluation study.

**S1b Table. Evaluation studies by the Italian Valutazione della Qualità della Ricerca VQR 2011-2014, published in 2017.**

| **ID** | **Evaluation object** | **EO** | **Research Field** | **Authors*** | **Titel of VQR ANVUR Report with Annexe** |
| --- | --- | --- | --- | --- | --- |
| A1b | Universites and non-university research in 10 disciplinary subfields | RO | Mathematics and Informatics | Abate M, Fusco N, Samarati P et al. | Rapporto finale di area Gruppo di Esperti della Valutazione dell’Area 01 – Scienze Matematiche e Informatiche (GEV01) |
| A2b | Universites and non-university research in 8 disciplinary subfields | RO | Physical Sciences | Zecchina R, Troncon C, Marinari V, Mattarese S, Guidi V, Bartolino R et al. | Rapporto finale di Area Gruppo di Esperti della Valutazione dell’Area 02 (GEV02) – Scienze Fisiche |
| A3b | Universites and non-university research in 12 disciplinary subfields | RO | Chemistry | Guerra G, Brandi A, Palleschi G, Russo N et al. | Rapporto finale di area Gruppo di Esperti della Valutazione dell’Area 3 (GEV03) – Chimica |
| A4b | Universites and non-university research in 12 disciplinary subfields | RO | Geosciences | Frezzotti M, et al. | Rapporto finale di area Gruppo di Esperti della Valutazione dell’Area 04 (GEV04) – Scienze della Terra |
| A5b | Universites and non-university research in 19 disciplinary subfields | RO | Biological Sciences | Tramontano A, Carnevali O, Schiepatti M, Bolognesi M, Parolaro D, et al. | Rapporto finale di Area Gruppo di Esperti della Valutazione dell’Area 05 (GEV05) – Scienze Biologiche |
| A6b | Universites and non-university research in 52 disciplinary subfields | RO | Medicine | Volpe M, Migliore L, Perticone F, Barbarisi A, Fineschi V, et al. | Rapporto finale di Area Gruppo di Esperti della Valutazione dell’Area 06 (GEV06) – Scienze Mediche |
| A7b | Universites and non-university research in 30 disciplinary subfields | RO | Agrarian and Veterinary Sciences | Varanini Z, Guglielmini C, et al. | Rapporto finale di area Gruppo di Esperti della Valutazione dell’Area 07 (GEV07) – Scienze agrarie e veterinarie |
| A8b | Universites and non-university research in 12 disciplinary subfields | RO | Engineering | Armanini A, et al. | Rapporto finale di area Gruppo di Esperti della Valutazione dell’Area Ingegneria (GEV08b)** |
| A9b | Universites and non-university research in 39 disciplinary subfields | RO | Industrial and Information Engineering | Setti G, Colombo MG, Perego P, Poncino F, et al. | Rapporto finale di area Gruppo di Esperti della Valutazione dell’Area 09 (GEV09) – Ingegneria Industriale e dell’Informazione |

**S1b Table continued.**

| **ID** | **Evaluation object** | **EO** | **Research Field** | **Authors*** | **Titel of VQR ANVUR Report with Annexe** |
| --- | --- | --- | --- | --- | --- |
| A10b | Universites and non-university research in 8 disciplinary subfields | RO | Psychology | Cubelli R, et al. | Rapporto finale di area Gruppo di Esperti della Valutazione dell’Area 11b (GEV11b) – Scienze Psicologiche |
| A11b | Universites and non-university research in 19 disciplinary subfields | RO | Economics and Statistics | Bertocchi G, Brugiavini A, Torrisi S, Bartolucci F, et al. | Rapporto finale di area Gruppo di Esperti della Valutazione dell’Area 13 (GEV13) – Scienze Economiche e Statistiche |

* Refers to the president and coordinators et al. of the expert groups leading the respective disciplinary evaluation study.

** The report of GEV08a Architecture was not included because of the small portion of products (0.14%) that were assessed bibliometrically.
