# Supplementary material for: Professional standards in bibliometric research evaluation? A meta-evaluation of European assessment practice 2005–2019
Source: PLoS One. 2020 Apr 20;15(4):e0231735. doi: 10.1371/journal.pone.0231735 (PMC7170233; doi:10.1371/journal.pone.0231735)
Supplement: S2 Table — (DOCX) [file pone.0231735.s002.docx]

**S2 Table: Evaluation studies by the Centre for Science and Technology Studies CWTS, Leiden 2005-2019.**

| **ID** | **Evaluation Object** | **EO** | **Research Field** | **CY** | **Authors*** | **PY** | **Title** | **Source** |
| --- | --- | --- | --- | --- | --- | --- | --- | --- |
| B1 | University of Amsterdam and Free University Amsterdam | RO | Physics | NL | van Leeuwen TN, Visser MS, van der Wurff LJ | 2005 | Physics research 1996-2002 Universiteit van Amsterdam and Vrije Universiteit Amsterdam | QANU Report |
| B2 | University of Amsterdam | RO | Ecology | NL | van Leeuwen TN, Calero Medina CM, Nederhof AJ | 2007 | Assessment of Research Quality. Institute for Biodiversity and Ecosystem Dynamics | University Report |
| B3 | University of Amsterdam | RO | Life Sciences | NL | van Leeuwen TN, Calero Medina CM, Nederhof AJ | 2007 | Assessment of Research Quality. Swammerdam Institute of Life Sciences | University Report |
| B4 | Lund University | RO | Multidisciplinary | SE | Noyons E | 2008 | Bibliometric Study of Lund University 2002-2007 | CWTS Report |
| B5 | Ten biomedical research centres funded by the English Ministry of Health NIHR | RO | Biomedical research | UK | Van Leeuwen TN, Hoorens S, Grant J | 2009 | Using bibliometrics to support the procurement of NIHR biomedical research centres in England | *Research Evaluation 18*(1): 71-82. |
| B6 | Netherlands Research School for Astronomy NOVA | RO | Astronomy | NL | Kamphuis P, van der Kruit PC | 2010 | Citations and impact of Dutch astronomy | http://arxiv.org/ abs/1011.5311 |
| B7 | KWR Watercycle Research Institute | RO | Water research | NL | n.a. | 2011 | Bibliometric Study on KWR Watercycle Research Institute | CWTS Report |
| B8 | Tampere University of Technology | RO | Multidisciplinary | FI | n.a. | 2011 | Tampere University of Technology research assessment exercise 2011. Bibliometric report 2005-2010. | University Report |
| B9 | University of Uppsala | RO | Multidisciplinary | SE | Nordgren J, Andersson P, Eriksson L, Sundquist B | 2011 | Quality and Renewal. An overall evaluation of research at Uppsala University | University Report |

**S2 Table continued**

| **ID** | **Evaluation Object** | **EO** | **Research Field** | **CY** | **Authors*** | **PY** | **Title** | **Source** |
| --- | --- | --- | --- | --- | --- | --- | --- | --- |
| B10 | KNAW-Institute NIOO | RO | Ecology | NL | Noyons E | 2012 | Bibliometric analysis of the research performance of NIOO-KNAW 2012 | CWTS Report |
| B11 | Faculty of Veterinary Medicine, University Utrecht | RO | Veterinary medicine | NL | Noyons E | 2012 | Results of the bibliometric study on the Faculty of Veterinary Medicine of the Utrecht University 2001-2010 | CWTS Report |
| B12 | KNAW-Institute ICIN | RO | Cardiology | NL | n.a. | 2012 | ICIN Netherlands Heart Institute. Self-evaluation 2005-2011 | ICIN Report |
| B13 | Research Councils of NWO | FI | Physical sciences, chemistry, geosciences | NL | Van Leeuwen TN, Moed HF | 2012 | Funding decisions, peer review, and scientific excellence in physical sciences, chemistry, and geosciences | *Research Evaluation 21*: 189-198. |
| B14 | FWF Austrian Science Fund | FI | Multidisciplinary | AT | van Wijk E, Costas-Comesana R | 2012 | Bibliometric Study of FWF Austrian Science Fund 2001-2010/11 | CWTS Report |
| B15 | Hanken School of Economics | RO | Economics | FI | Liljeblom E | 2012 | Hanken School of Economics. Evaluation of Research | University Report |
| B16 | University of Helsinki | RO | Multidisciplinary | FI | Saari S, Moilanen A (eds.) | 2012 | International evaluation of research and doctoral training at University of Helsinki 2005-2010 | University Report |
| B17 | Public research in Luxemburg | RO | Multidisciplinary | LX | Noyons E | 2012 | Luxembourg bibliometric report CWTS/FNR 2012 | Report by CWTS & Fond National de la Recherche FNR |
| B18 | Research Council of Norway | FI | Multidisciplinary | NO | Van Leeuwen TN | 2012 | Evaluation of the Research Council of Norway. Background Report No. 6 - | CWTS Report |

**S2 Table continued**

| **ID** | **Evaluation Object** | **EO** | **Research Field** | **CY** | **Authors*** | **PY** | **Title** | **Source** |
| --- | --- | --- | --- | --- | --- | --- | --- | --- |
| B19 | DNRF Excellence Centres at Universities | FI | Multidisciplinary | DK | Schneider JW, Costas R | 2013 | Bibliometric analyses of publications from Centres of Excellence funded by the Danish National Research Foundation. | Report by CWTS with Danish Centre for Studies in Research and Research Policy, Aarhus University |
| B20 | KNAW-Institute CBS | RO | Fungal biodiversity | NL | Costas-Comesana R | 2014 | Self-evaluation report 2008–2013 of The CBS Fungal Biodiversity Centre (CBS-KNAW) | CBS Report |
| B21a | Dutch Academic Medical Centers | RO | Medicine | NL | Van Leeuwen TN | 2014 | Bibliometric study on Dutch academic medical centers | CWTS Report |
| B22 | Anonymous European Research Council | FI | Multidisciplinary | na | Calero Medina C, van Wijk E | 2014 | Analyses of the scholarly and scientific output from grants funded by the “Anonymous Research Council” from 2005 to 2008 | Bibliometric Annex to Anonymous Agency Report |
| B23 | University of Helsinki | RO | Multidisciplinary | FI | Forsman M, Nane T, Noyons E | 2014 | Research performance analysis for the University of Helsinki 2005-2012/13 | CWTS Report |
| B24 | Mälardalen University | RO | Multidisciplinary | SE | Noyons E, Nane T | 2014 | Bibliometric report of Mälardalen University | CWTS Report |
| B25 | NHS Health technology assessment programme | FI | Medicine, clinical research | UK | Guthrie S, Bienkowska-Gibbs T, Manville C, Pollitt A, Kirtley A, Wooding S | 2015 | The impact of the National Institute for Health Research Health Technology Assessment programme, 2003–13: a multimethod evaluation | Health Technology Assessment 19 (67) |
| B26 | Academy of Finland’s Centre of Excellence Programmes | FI | Multidisciplinary | FI | Nuutinen A, Gyran M, Kosten J, Waltman L | 2015 | Bibliometric impact analysis of the Academy of Finland’s Centre of Excellence Programmes | CWTS Report |

**S2 Table continued**

| **ID** | **Evaluation Object** | **EO** | **Research Field** | **CY** | **Authors*** | **PY** | **Title** | **Source** |
| --- | --- | --- | --- | --- | --- | --- | --- | --- |
| B27 | Centre of Human Drug Research | RO | Economics | NL | Kosten J | 2015 | Bibliometric analysis of the Performance of the Centre for Human Drug Research 2001-2013/14 | CWTS Report |
| B28 | Candidates for joining anonymous European university network | RO | Multidisciplinary | EU | Van Wijk E | 2016 | Benchmark analysis of “network” candidate universities 2016-2020 | CWTS Report |
| B29 | Synchrotron radiation facility of Diamond Light Source | RO | Synchrotron radiation | UK | Noyons E, van Wijk E | 2016 | Bibliometric performance measurement of Diamond Light Source research 2007-2014 | CWTS Report |
| B30 | Anonymous Dutch university | RO | Multidisciplinary | NL | Van Leeuwen T, van Wijk E, Wouters P | 2016 | Bibliometric analysis of output and impact based on CRIS data: a case study on the registered output of a Dutch university | Scientometrics 106: 1–16 |
| B21b | Dutch university medical centres | RO | Medicine, cardiovascular research | NL | Van Welie SD, van Leeuwen TN, Bouma CJ, Klaassen ABM | 2016 | The joint cardiovascular research profile of the university medical centres in the Netherlands | Neth Heart J 24: 308–316 |
| B31 | Health research board Ireland | FI | Medicine, Life Sciences | IE | Kosten J, Hiney M | 2017 | A bibliometric analysis of research publication output supported by the health research board (2013-16) | CWTS Report |
| B32 | RIVM SOR programme | RO | Environmental medicine | NL | Van Wijk E, Meijer I | 2017 | Research performance analysis for the SOR programme of the Rijksinstituut voor Volksgezondheid en Milieu (RIVM) 2011-2014/15 | CWTS Report |
| B33 | University of Groningen, Faculty of Economics & Business | RO | Economics and business | NL | Neijssel M, Ferreira Goncalves M | 2018 | Bibliometric performance of the Faculty of Economics & Business (FEB) (2008-2016) | CWTS Report |

**S2 Table continued**

| **ID** | **Evaluation Object** | **EO** | **Research Field** | **CY** | **Authors*** | **PY** | **Title** | **Source** |
| --- | --- | --- | --- | --- | --- | --- | --- | --- |
| B34a | Gothenburg university | RO | Multidisciplinary | SE | Jarvening Bo | 2018 | Bibliometriska analyser: Annex 2018. Göteborgs universitet 2012-2016 | Göteborg Universitetsbibliotek Report |
| B34b | Gothenburg university | RO | Multidisciplinary | SE | Jarvening Bo | 2019 | Bibliometriska analyser: Annex 2019. Göteborgs universitet 2013-2017 | Göteborg Universitetsbibliotek Report |
| B35 | Swiss Federal Laboratories for Materials Science and Technology | RO | Materials science and technology | CH | Kosten J | 2019 | Bibliometric Study of Empa | CWTS Report |
| B36 | Anonymous German University | RO | Multidisciplinary | DE | Costas R | 2019 | Bibliometric benchmark analysis: “Anon.” University 2008-2016 | CWTS Report |
| B37 | European Association of Research and Technology Organisations EARTO | RO | Multidisciplinary | EU | Noyons E, Ferreira M, de Bordes C | 2019 | Research performance assessment of EARTO-8, using bibliometrics | CWTS Report |

* Refers to authors of the bibliometric analyses where possible, otherwise to authors or editors of more comprehensive evaluation reports.
