# Supplementary material for: Professional standards in bibliometric research evaluation? A meta-evaluation of European assessment practice 2005–2019
Source: PLoS One. 2020 Apr 20;15(4):e0231735. doi: 10.1371/journal.pone.0231735 (PMC7170233; doi:10.1371/journal.pone.0231735)
Supplement: S3 Table — (DOCX) [file pone.0231735.s003.docx]

**S3 Table. Evaluation studies by the Nordic Institute for Studies in Innovation, Research and Education NIFU, Oslo, 2005-2019.**

| **ID** | **Evaluation Object** | **EO** | **Research Field** | **CY** | **Authors*** | **PY** | **Title** | **Source** |
| --- | --- | --- | --- | --- | --- | --- | --- | --- |
| C1 | Physics departments and research groups at 11 Norwegian institutions | RO | Physics | NO | Aksnes DW | 2010 | Evaluation of Physics Research in Norway. Bibliometric Analysis | NIFU STEP/ Research Council of Norway Report |
| C2 | 15 earth sciences departments and research groups at 8 Norwegian universities plus groups at 10 research institutions | RO | Earth sciences | NO | Aksnes DW, Klitkou A | 2011 | Research in Earth sciences in Norway. Bibliometric analysis | NIFU STEP/ Research Council of Norway Report |
| C3 | 8 mathematics departments and units at 7 Norwegian universities | RO | Mathematics | NO | Aksnes DW | 2012 | Research in mathematics at Norwegian universities. Bibliometric analysis | NIFU STEP/ Research Council of Norway Report |
| C4 | 18 mathematics departments and units at 10 Norwegian universities and 3 research institutes | RO | Information and communication technology | NO | Aksnes DW | 2012 | Research in information and communication technology in Norway. Bibliometric analysis | NIFU STEP/ Research Council of Norway Report |
| C5 | 204 out of a total of 287 national centres of excellence in four Nordic countries | FI | Multidisciplinary | DK, FI, NO, SE | Aksnes DW, Benner M, Borlaug SB, Hansen HF, Kallerud E, Kristiansen E, Langfeldt L, Pelkonen A, Sivertsen G | 2012 | \| Centres of excellence in the Nordic countries. A comparative study of research excellence policy and excellence centre schemes in Denmark, Finland, Norway and Sweden \| \| --- \| | NIFU working paper 4/12 |

**S3 Table continued**

| **ID** | **Evaluation Object** | **EO** | **Research Field** | **CY** | **Authors*** | **PY** | **Title** | **Source** |
| --- | --- | --- | --- | --- | --- | --- | --- | --- |
| C6 | 11 selected centres of excellence from four Nordic countries before and after introduction of excellence scheme | FI | Multidisciplinary | DK, FI, NO, SE | Langfeldt L, Borlaug SB, Aksnes DW, Benner M, Hansen HF, Kallerud E, Kristiansen E, Pelkonen A, Sivertsen G | 2013 | Excellence initiatives in Nordic research policies. Policy issues – tensions and options | NIFU working paber 10/13 |
| C7 | University of Bergen | RO | Marine sciences | NO | Aksnes DW | 2014 | Evaluation of the strategic priority area of marine research and education at the University of Bergen | Report of the evaluation panel to the University |
| C8 | 20 engineering departments and research groups at 9 Norwegian universities and university collges plus groups at 8 research institutes | RO | Engineering science | NO | Aksnes DW | 2015 | Basic and long-term research within Engineering Science in Norway. Bibliometric analysis | NIFU STEP/ Research Council of Norway Report |
| C9 | Simula research laboratory | RO | Computer Science | NO | Aksnes DW | 2016 | Simula research laboratory. Publication analysis 2009-2015 | NIFU working paper 4/16 |
| C10 | 172 Higher education institutions in five Nordic countries | RO | Multidisciplinary | DK, FI, IS, NO, SE | Piro FN (ed) | 2017 | Comparing research at Nordic higher education institutions using bibliometric indicators covering the years 1999-2014 | Nordforsk Policy Paper 4/17 |
| C11 | 6 HEs and 5 institutes in Norway | RO | Polar research and research in Svalbard | NO | Aksnes DW | 2017 | Norwegian Polar Research & Svalbard Research. Publication analysis | NIFU working paper 6/17 |
| C12 | University of Bergen | RO | Multidisciplinary | NO | Aksnes DW | 2018 | Publiseringsanalyse – Universitetet i Bergen. Faglig profil og siteringshyppighet | Arbeidsnotat 2018:3 |

* Refers to authors of the bibliometric analyses where possible, otherwise to authors or editors of more comprehensive evaluation reports.
