# Supplementary material for: Professional standards in bibliometric research evaluation? A meta-evaluation of European assessment practice 2005–2019
Source: PLoS One. 2020 Apr 20;15(4):e0231735. doi: 10.1371/journal.pone.0231735 (PMC7170233; doi:10.1371/journal.pone.0231735)
Supplement: S4 Table — (DOCX) [file pone.0231735.s004.docx]

**S4 Table. Evaluation studies with bibliometric analysis by Thomson Reuters Evidence Ltd/ Clarivate Analytics, London, 2005-2019.**

| **ID** | **Evaluation Object** | **EO** | **Research Field** | **CY** | **Authors*** | **PY** | **Title** | **Source** |
| --- | --- | --- | --- | --- | --- | --- | --- | --- |
| D1 | Wellcome Trust | FI | Human genetics | UK | Allen L, Dunn M, Vaughan C | 2010 | Human genetics 1990-2009 | Wellcome Trust Report „Portfolio Review“ |
| D2 | Wellcome Trust | FI | Functional brain imaging | UK | Vaughan C, Allen L | 2011 | Human functional brain imaging 1990-2009 | Wellcome Trust Report „Portfolio Review“ |
| D3 | Wellcome Trust | FI | Medicine | UK | Allen L et al. | 2012 | Assessment framework report. Volume 1: Report 2011/2012 | Wellcome Trust Report „Assessment framework reports“ |
| D4 | Wellcome Trust | FI | Medicine | UK | Allen L et al. | 2013 | Assessment framework report. Volume 1: Report 2011/2012 | Wellcome Trust Report „Assessment framework reports“ |
| D5 | Medical Research Council MRC funded research | FI | Medicine | UK | Reddick G, Charman E | 2014 | Outputs, outcomes and impact of MRC research: 2013/14 report | Medical Research Council Report |
| D6 | Health Research Board | FI | Medicine, biological sciences | IE | Thomson Reuters Evidence | 2014 | Bibliometric analysis of HRB-supported publications 2000-12 | Health Research Board Report |
| D7 a-j* | Innovative Medicines Initiative funded projects | FI | Medicine | EU | Thomson Reuters/ Clarivate Analytics | 2012-2019 | Bibliometric analysis of ongoing projects 1^st^-10^th^ report | Innovative Medicines Initative Reports |

* Ten consecutive monitoring reports with identical methods are treated as one study (one analysed item).
