# Supplementary material for: Professional standards in bibliometric research evaluation? A meta-evaluation of European assessment practice 2005–2019
Source: PLoS One. 2020 Apr 20;15(4):e0231735. doi: 10.1371/journal.pone.0231735 (PMC7170233; doi:10.1371/journal.pone.0231735)
Supplement: S5 Table — (DOCX) [file pone.0231735.s005.docx]

**S5 Table. Evaluation studies by the Spanish Council for Scientific Research CSIC, 2005-2019.**

| **ID** | **Evaluation Object** | **EO** | **Research Field** | **CY** | **Authors*** | **PY** | **Title** | **Source** |
| --- | --- | --- | --- | --- | --- | --- | --- | --- |
| E1 | CSIC | RO | Natural resources and environmental sciences | ES | Costas R, Bordons M | 2005 | Bibliometric indicators at the micro-level: some results in the area of natural resources at the Spanish CSIC | *Research Evaluation 14*(2): 110-120 |
| E2 | CSIC | RO | Biotechnology | ES | Albert A, Granadino B, Plaza LM | 2007 | Scientific and technological performance evaluation of the CSIC in the field of biotechnology | *Scientometrics 70*(1): 41-51 |
| E3 | CSIC | RO | Multidisciplinary | ES | González-Albo B, Moreno L, Morillo F, Bordons M | 2012 | Bibliometric indicators for the analysis of the research performance of a multidisciplinary institution: the CSIC | *Revista Española de Documentación Científica 35*(1): 9-37 |
| E4a-i* | CSIC | RO | Multidisciplinary | ES | Bordons M, Morillo F, Gomez I, Moreno L, Aparicio J, González-Albo B | 2011-2018 | La actividad científica del CSIC a través de indicadores bibliométricos (Web of Science, consecutive five year periods, 2006-2017) | CSIC report series |
| E5 | CSIC | RO | Social sciences, humanities | ES | Diaz-Fez AA, Bordons M, van Leeuwen T | 2016 | Integrating metrics to measure research performance in social sciences and humanities: The case of the Spanish CSIC | *Studies in Higher Education 41*(11): 2044–2060 |

* Nine consecutive reports with identical methods are treated as one study (one analysed item).
