# Supplementary material for: Professional standards in bibliometric research evaluation? A meta-evaluation of European assessment practice 2005–2019
Source: PLoS One. 2020 Apr 20;15(4):e0231735. doi: 10.1371/journal.pone.0231735 (PMC7170233; doi:10.1371/journal.pone.0231735)
Supplement: S6 Table — (DOCX) [file pone.0231735.s006.docx]

**S6 Table. Evaluation studies of research organisations by other bibliometric experts 2005-2019.**

| **ID**** | **Evaluation Object** | **EO** | **Research Field** | **CY** | **Authors*** | **PY** | **Title** | **Source** |
| --- | --- | --- | --- | --- | --- | --- | --- | --- |
| F1 | 35 public medical faculties in Germany | RO | Medicine | DE | Grohmann G, Stegmann J | 2005 | German medical faculties in the 1990s: on-line bibliometric analysis | *Research Evaluation 14*(2): 157-164 |
| F2 | 13 Hungarian Academy of Science Institutes | RO | Physics, chemistry, mathematics, life sciences | HU | Vinkler P | 2006 | Composite scientometric indicators for evaluating publications of research institutes | *Scientometrics 68*(3): 629-643 |
| F3 | Netherlands Research School for Environmental Socio-economic and natural sciences (SENSE) | RO | Environmental social and natural sciences | NL | Gerritsma W, Duizendstraal MB, Fransen H, Loman ME | 2007 | Bibliometric analysis of the Netherlands Research School for the Socio-Economic and Natural Sciences of the Environment (SENSE) | Wageningen University Research Library Report |
| F4a | 77 Italian universities | RO | Multidisciplinary (disciplinary areas as used in IDA1-A9) | IT | Abramo G, D´Angelo CA, Caprasecca A | 2009 | Allocative efficiency in public research funding: Can bibliometrics help? | *Research Policy 38:* 206-215 |
| F4b | 69 Italian universities | RO | Multidisciplinary (see above) | IT | Abramo G, D´Angelo CA | 2009 | Assessing technical and cost efficiency of research activities: a case study of the Italian university system | *Research Evaluation 18*(1): 61-70 |

**S6 Table continued**

| **ID**** | **Evaluation Object** | **EO** | **Research Field** | **CY** | **Authors*** | **PY** | **Title** | **Source** |
| --- | --- | --- | --- | --- | --- | --- | --- | --- |
| F5 | Universities of Vienna in comparison to Zurich and Oslo | RO | Social Sciences | AT, CH, NO | Gorraiz J, Greil M, Mayer W, Reimann R, Schiebel E | 2009 | International publication output and research impact in social sciences: comparison of the Universities of Vienna, Zurich, and Oslo | *Research Evaluation 18*(3): 221-232 |
| F6 | Medical Faculties and University Hospitals in Finland and Sweden | RO | Medicine | FI, SE | Karlsson S, Jonsson A | 2009 | Clinical research in Finland and Sweden. Evaluation Report | Publications ft he Academy of Finland 5/09 |
| F7 | Aalto university | RO | Multidisciplinary | FI | Sandström U | 2009 | Aalto university bibliometric report | Aalto university project report |
| F8 | Anonymous Swedish University | RO | Multidisciplinary | SE | Sandström U | 2009 | Research quality and diversity of funding: A model for relating research money to output of research | *Scientometrics 79*(2): 341-349 |
| F9 | University of Stockholm, 20 natural science departments | RO | Multidisciplinary | SE | Colliander C | 2011 | The effects and their stability of field normalization baseline on relative performance with respect to citation impact: A case study of 20 natural science departments | *Journal of Informetrics 5*: 101-113 |
| F10 | Research groups at 17 universities | RO | Production technology and manufacturing systems | IT | Franceschini F, Maisano D | 2011 | Structured evaluation of the scientific output of academic research groups by recent h-based indicators | *Journal of Informetrics 5*: 64-74 |
| F11 | 104 scientists at 23 UK university departments | RO | Medicine, anaesthesia | UK | Moppett IK, Hardman JG | 2011 | Bibliometrics of anaesthesia researchers in the UK | *British journal of anaesthesia 107*(3): 351-6 |
| F12 | UK cancer centres | RO | Medicine, oncology | UK | Sullivan R, Lewison G, Purushotham AD | 2011 | An analysis of research activity in major UK cancer centres | *European Journal of Cancer 47*: 536-544 |

**S6 Table continued**

| **ID**** | **Evaluation Object** | **EO** | **Research Field** | **CY** | **Authors*** | **PY** | **Title** | **Source** |
| --- | --- | --- | --- | --- | --- | --- | --- | --- |
| F13 | Engineering departments at four GR universities | RO | Engineering | GR | Vaxevanidis NM, Despotidi H, Prokopiou H, Koutsomichalis A | 2011 | On the evaluation of the quality of research in Greek HEIs using bibliometric indices | *International Journal for Quality Research 5*(4), 247-254 |
| F14 | 7 Economic Institutes of the Leibniz Gemeinschaft | RO | Economics | DE | Ketzler R, Zimmermann KF | 2012 | A citation analysis of economic research institutes | *Scientometrics 95*: 1095-1112 |
| F15 | Deutsches Rheuma Forschungszentrum DRFZ | RO | Medicine, rheumatology | DE | Pudovkin A, Kretschmer H, Stegmann J, Garfield E | 2012 | Research evaluation. Part I: productivity and citedness of a German medical research institution | *Scientometrics 93*: 3-16 |
| F16 | University faculties in the Netherlands | RO | Pedagogics and educational sciences | NL | Prins A | 2013 | Research Review Pedagogics and Education Sciences | Quality Assurance Netherlands Universities QANU Report |
| F17 | University of Antwerpen | RO | Natural sciences | BE | Engels TCE, Goos P, Dexters N, Spruyt EHJ | 2013 | Group size, h-index, and efficiency in publishing in top journals explain expert panel assessments of research group quality and productivity | *Research Evaluation 22*: 224-236 |
| F18 | University faculties in the Netherlands | RO | Cultural Anthropology | NL | Prins A | 2014 | Research Review Cultural Anthropology 2007-2012 | QANU Report |
| F19 | Methodological recommendations to Max Planck Society MPG | RO | Natural sciences | DE | Bornmann L, Marx W | 2014 | How to evaluate researchers working in the natural and life sciences meaningfully? A proposal based on percentiles of citations | *Scientometrics 98*: 487-509 |
| F20 | Max Planck Institute for Chemistry | RO | Chemistry | DE | Haunschild R, Schier H, Bornmann L | 2014 | Max Planck Institute for Chemistry. Scientific report 2012-2014 | MPI for Chemistry Institute report |

**S6 Table continued**

| **ID**** | **Evaluation Object** | **EO** | **Research Field** | **CY** | **Authors*** | **PY** | **Title** | **Source** |
| --- | --- | --- | --- | --- | --- | --- | --- | --- |
| F21 | Medical Schools in Greece | RO | Medicine | GR | Kazakis NA, Diamantidis AD, Fragidis LL, Lazarides MK | 2014 | Evaluating the research performance of the Greek medical schools using bibliometrics | *Scientometrics 98*: 1367–1384 |
| F22 | Civil engineering departments at five institutions | RO | Civil engineering | GR | Kazakis NA | 2014 | Bibliometric evaluation of the research performance of the Greek civil engineering departments in National and European context | *Scientometrics 101*: 505-525 |
| F23 | Chemical engineering departments at three institutions | RO | Chemical engineering | GR | Kazakis NA | 2015 | The research activity of the current faculty of the Greek chemical engineering departments: a bibliometric study in national and international context | *Scientometrics 103: 229–250* |
| F24 | 37 neurosurgical departments in UK and Ireland | RO | Medicine, neurosurgery | UK, IE | Knight J | 2015 | Academic impact rankings of neurosurgical units in the UK and Ireland, as assessed with the h-index | *British Journal of Neurosurgery 29(5):* 637-643 |
| F25a | 64 political science, sociology, and marketing departments in Romania | RO | Social sciences, marketing | RO | Miroiu A, Păunescu M, Vîiu GA | 2015 | Ranking Romanian academic departments in three fields of study using the g-index | *Quality in Higher Education 21(2):* 189-212 |
| F26a | Departments in four disciplines in UK | RO | Biology, Chemistry, Physics, Sociology | UK | Mryglod O, Kenna R, Holovatch Y, Berche B | 2015 | Predicting results of the Research Excellence Framework using departmental h-index | *Scientometrics 102:* 2165–2180 |
| F26b | Departments in four disciplines in UK | RO | Biology, Chemistry, Physics, Sociology | UK | Mryglod O, Kenna R, Holovatch Y, Berche B | 2015 | Predicting results of the research excellence framework using departmental h-index: revisited | *Scientometrics 104:* 1013–1017 |

**S6 Table continued**

| **ID**** | **Evaluation Object** | **EO** | **Research Field** | **CY** | **Authors*** | **PY** | **Title** | **Source** |
| --- | --- | --- | --- | --- | --- | --- | --- | --- |
| F27 | University of Belgrade | RO | Multidisciplinary | RS | Zornic N, Bornmann L et al. | 2015 | Ranking institutions within a university based on their scientific performance: A percentile-based approach | *El profesional de la información 24(5):* 551-566 |
| F28 | Adult social care research in the UK | RO | Social care research | UK | Campbell D, Côté G, Grant J, Knapp M, Mehta A, Jones MM | 2016 | Comparative Performance of Adult Social Care Research | *British Journal of Social Work 46:* 1282–1300 |
| F29 | Aarhus university | RO | Multidisciplinary | DK | Nielsen MW | 2016 | Gender inequality and research performance: moving beyond individual-meritocratic explanations of academic advancement | *Studies in Higher Education 41(11):* 2044–2060 |
| F30 | ISIS Neutron and Muon Source at the Rutherford Appleton Laboratory | RO | Physical and life sciences | UK | Simmonds P, Brown N, Rosemberg C, Varnai P, Rental m, Koc Y, Angelis J, Kosk K | 2016 | ISIS Lifetime Impact Study - Volume 1; Appendix E Bibliometric analysis of ISIS papers | Technopolis Report in collaboration with STFC, Nov. 2016 |
| F25b | Universities in Romania | RO | Multidisciplinary | RO | Vîiu GA, Păunescu M, Miroiu A | 2016 | Research-driven classification and ranking in higher education: an empirical appraisal of a Romanian policy experience | *Scientometrics 107:* 785–805 |
| F31 | 21 universities in Spain | RO | Medicine, dentistry | ES | De la Flor-Martinez et al. | 2017 | Evaluation of scientific output in Dentistry in Spanish Universities | *Med Oral Patol Oral Cir Bucal. 22 (4):* e491-9. |
| F32 | 130 universities in the UK | RO | Multidisciplinary | UK | Mingers J, O’Hanley JR, Okunola M | 2017 | Using Google Scholar institutional level data to evaluate the quality of university research | *Scientometrics 113:* 1627–1643 |

**S6 Table continued**

| F33 | 14 economics faculties in Slovakia | RO | Economics | SK | Ciaian P, Lancaric D, Pokrivcak J | 2018 | The Productivity of Economics Research in Slovakia | *Eastern European Economics 56(2): 168-200* |
| --- | --- | --- | --- | --- | --- | --- | --- | --- |
| F34 | University Medical Faculties in Romania | RO | Medicine | RO | Liu X, Păunescu M, Proteasa V, Wu J | 2018 | Minimum Representative Size in Comparing Research Performance of Universities: the Case of Medicine Faculties in Romania | *Journal of Data and Information Science 3(3):* 32-42 |
| F35 | CSIC and four European benchmark organizations | RO | Multidisciplinary | ES | Maldonado Carillo J, Martinez M | 2018 | Análisis bibliométrico comparativo de la actividad científica del CSIC y cuatro homólogos europeos: CNRS, HG, MPG Y CNR (2006-2015) | *Revista General de Información y Documentación 28(1):* 163-191 |
| F36 | 50 Departments in Greece | RO | Science and Engineering | GR | Pitsolanti M, Papadopoulou F, Tselios N | 2018 | A Scientometric Evaluation of 50 Greek Science and Engineering University Departments using Google Scholar | *Journal of Scientometric Res. 7(1):* 09-18 |
| F37 | 47 university anästhesiology clinics in Germany, Austria, and Switzerland | RO | Medicine anaesthesia | DE, AT, CH | Miller et al. | 2019 | Anästhesiologie-Publikationen aus Deutschland, Österreich und der Schweiz 2011–2015 | *Anaesthesist 68*: 294–302 |
| F38 | 34 universities in Germany | RO | Medicine, cardiac surgery, general surgery, and cardiology | DE | Schwarzer M, Alscher L, Doenst T | 2019 | Comparison of Scientific Publications from Three Different Clinical Disciplines of German Universities | *Thoracic and Cardiovascular Surgeon 67(6):* 488-493 |

* Refers to authors of the bibliometric analyses, where possible, otherwise to authors or editors of more comprehensive evaluation reports.

** Some evaluation studies are covered by more than one publication, sometimes in consecutive years. As a consequence, the number of all publications (n=41) documented here is larger than the total number of evaluation studies (n=38). Related publications carry the same ID and are distinguished by a, b, c (first row).
