# Supplementary material for: Professional standards in bibliometric research evaluation? A meta-evaluation of European assessment practice 2005–2019
Source: PLoS One. 2020 Apr 20;15(4):e0231735. doi: 10.1371/journal.pone.0231735 (PMC7170233; doi:10.1371/journal.pone.0231735)
Supplement: S7 Table — (DOCX) [file pone.0231735.s007.docx]

**S7 Table: Evaluation studies of funding instruments by other bibliometric experts 2005-2019.**

| **ID**** | **Evaluation Object** | **EO** | **Research Field** | **CY** | **Authors*** | **PY** | **Title** | **Source** |
| --- | --- | --- | --- | --- | --- | --- | --- | --- |
| G1a | European Molecular Biology Organization EMBO Longterm Fellowships and Young Investigator Programme | FI | Life Sciences | DE | Ledin A, Bornmann L, Gannon F, Wallon G | 2007 | A persistent problem. Traditional gender roles hold back female scientists | *EMBO reports 8*(11): 982-987 |
| G1b | EMBO Longterm Fellowships and Young Investigator Programme | FI | Life Sciences | DE | Bornmann L, Wallon G, Ledin A | 2008 | Does the committee peer review select the best applicants for funding? An investigation of the selection process for two EMBO programmes | *PLoS One 3*(10): e3480 |
| G1c | EMBO Longterm Fellowships and Young Investigator Programme | FI | Life Sciences | DE | Bornmann L, Wallon G, Ledin A | 2008 | Is the h index related to (standard) bibliometric measures and to the assessments by peers? An investigation of the h index by using molecular life sciences data | *Research Evaluation 17*(2): 149-156. |
| G2a | Deutsche Forschungsgemeinschaft DFG Emmy Noether Programm | FI | Medicine, physics, chemistry, biology | DE | Böhmer S, Hornbostel S, Meuser M | 2008 | Postdocs in Deutschland: Evaluation des Emmy Noether-Programms | IFQ-Working Paper No. 3 |
| G2b | DFG Emmy Noether Programm | FI | Medicine, physics | DE | Hornbostel S, Böhmer S, Klingsporn B, Neufeld J, von Ins M | 2009 | Funding of young scientists and scientific excellence | *Scientometrics 79*(1): 171-190. |
| G2c | DFG Emmy Noether Programm | FI | Multidisciplinary | DE | Böhmer S, Von Ins M | 2009 | Different - not just by label: research-oriented academic careers in Germany | *Research Evaluation 18*(3): 177-184 |

**S7 Table continued**

| **ID**** | **Evaluation Object** | **EO** | **Research Field** | **CY** | **Authors*** | **PY** | **Title** | **Source** |
| --- | --- | --- | --- | --- | --- | --- | --- | --- |
| G3 | 6th EU Framework Programme, all RTD Project Lead Scientists | FI | Multidisciplinary | EU | Larrue P, Cadiou Y, Laurens P, Arnold E | 2009 | Bibliometric profiling of Framework Programme participants | Technopolis; Final Report of Tender RTD.A.3/ MF/lb D(2007)D/ 549200 |
| G4 | 6th EU Framework Programme Subpriority "Global Change and Ecosystems" | FI | Environmental sciences, geosciences, water resources | EU | Arnold E | 2009 | Ex-Post Impact Assessment FP6 Subpriority "Global Change and Ecosystems" | Technopolis Report, published by EC Directorate General for Research –Directorate I Environment |
| G5 | Swedish Environmental Protection Agency SEPA research programs | FI | Environmental research | SE | Sandström U | 2009 | Bibliometric evaluation of research programs - A study of scientific quality | Swedish Environmental Protection Agency Report |
| G6 | Swedish Research Council VR | FI | Biodiversity research | SE | Karlsson S | 2010 | Evaluation of Swedish biodiversity research | Vetenskapsradet rapportserie 14: 2010 |
| G7 | 5th EU Framework Programme for the social sciences and humanities (SSH) | FI | Social sciences and humanities research | EU | Peter V, Leon LR, Cadiou Y, Doussineau M | 2010 | Evaluation of the Impact of Framework Programme supported Social Sciences and Humanities Research | Technopolis Report, published by EC Directorate General for Research – Directorate L Science Economy and Society RTD reports |
| G8a | European Research Council ERC Starting Grants | FI | Multidisciplinary | EU | Neufeld J, Huber N, Wegner A | 2012 | On the relationship between peer review-based selection decisions in individual research funding and applicants' publication performance: the case of the ERC starting grants | Proceedings of 17th International Conference on STI, Montréal: Science-Metrix and OST, Volume 2, 619-627 |

**S7 Table continued**

| **ID**** | **Evaluation Object** | **EO** | **Research Field** | **CY** | **Authors*** | **PY** | **Title** | **Source** |
| --- | --- | --- | --- | --- | --- | --- | --- | --- |
| G8b | European Research Council ERC Starting Grants | FI | Multidisciplinary | EU | Neufeld J, Hornbostel S | 2012 | Funding programmes for young scientists - do the 'best' apply? | *Research Evaluation 21*: 270-279 |
| G8c | European Research Council ERC Starting Grants | FI | Multidisciplinary | EU | Neufeld J, Huber N, Wegner A | 2013 | Peer-review-based selection decisions in individual research funding, applicants publication strategies and performance: The case of the ERC starting grants. | *Research Evaluation 22*: 237-247 |
| G9 | Danish Council for Strategic Research NABIIT Programme | FI | Nanotechnology, biotechnology, information and communication Technology | DK | DAMVAD | 2013 | A bibliometric analysis of publications from NABIIT projects | Danish Agency for Science,Technology and Innovation Report, Bibliometric Annex |
| G10 | Spanish National Health System „Miguel Servet Research Contract Programme“ | FI | Medicine | ES | Antonio-García M, López-Navarro I, Rey-Rocha J | 2014 | Determinants of success for biomedical researchers: a perception-based study in a health science research environment | *Scientometrics 101:* 1747-1779 |
| G11 | Austrian Science Fund FWF Erwin Schröder Fellowships | FI | Multidisciplinary | AT | Meyer N, Bührer S | 2014 | Impact Evaluation of the Erwin Schrödinger Fellowships with Return Phase | Frauhofer Institute Systems and Innovation Research Report |
| G12 | Seven Swedish Environmental Protection Agency SEPA research programs | FI | Environmental research | SE | Sandström U | 2014 | Bibliometric evaluation of SEPA-funded large research programs 2003-2013 | Swedish Environmental Protection Agency Report |

**S7 Table continued**

| **ID**** | **Evaluation Object** | **EO** | **Research Field** | **CY** | **Authors*** | **PY** | **Title** | **Source** |
| --- | --- | --- | --- | --- | --- | --- | --- | --- |
| G13 | University of Cambridge Neuroscience Initiative | FI | Neuroscience | UK | Gunashekar S, Lavoie R, Roberge G, Rashid M, Marjanovic S | 2015 | A bibliometric analysis of research by the Cambridge Neuroscience Strategic Research Initiative. Extended Summary | Rand Europe Report in collaboration with Science Metrix |
| G14 | ERC funded projects | FI | Multidisciplinary | EU | Robitaille J-P, Macaluso B, Pollitt A, Gunashekar S, Larivière V | 2015 | Comparative scientometric assessment of the results of ERC funded projects. Bibliometric assessment report (D5) | Report by Observatoire des sciences et des technologies (OST) and RAND Europe for European Research Council Executive Agency |
| G15a | Strategic Research Area Initiative by Swedish Research Council and other agencies | FI | Multidisciplinary | SE | Teeri T et al. | 2015 | Evaluation of the Strategic Research Area Initiative 2010-2014 | Swedish Research Council Report |
| G15b | Bibliometric assessment at Swedish Research Council | FI | Multidisciplinary | SE | Sjöstedt E, Aldberg H, Jacobbson C | 2015 | Guidelines for using bibliometrics at the Swedish Research Council | Vetenskapsradet  *Ref. No.* 113-2014-7357 |
| G16 | DFG excellence initiative | FI | Multidisciplinary | DE | Möller T, Schmidt M, Hornbostel S | 2016 | Assessing the effects of the German Excellence Initiative with bibliometric methods | *Scientometrics 109:* 2217–2239 |
| G17 | FP7 Project Danube basin Capacity building and excellence in river systems | FI | Environmental sciences | EU | Sidoroff M, Paraschiv M, Amarioarei A, Paun M | 2016 | Measuring funded research performance for multidisciplinary research in the Danube basin | *Journal of Environmental Protection and Ecology 17* (2): 638–647 |

**S7 Table continued**

| **ID**** | **Evaluation Object** | **EO** | **Research Field** | **CY** | **Authors*** | **PY** | **Title** | **Source** |
| --- | --- | --- | --- | --- | --- | --- | --- | --- |
| G18 | RDI programme “Smart Specialisation Strategy” | FI |  | LT | Dunauskas S, Jaujininkas V, Lapienis J, Reimeris R, Vytautas V | 2017 | Smart Specialisation Strategy Progress, First Report | MOSTA Report; project code 10.1.1-ESFA-V-912-01-0003 |
| G19 | Momentum grant | FI | Multidisciplinary | HU | Györffy B, Nagy AM, Herman P, Törö A | 2018 | Factors influencing the scientific performance of Momentum grant holders... | *Scientometrics 117*: 409–426 |

* Refers to authors of the bibliometric analyses where possible, otherwise to authors or editors of more comprehensive evaluation reports.

** Some evaluation studies are covered by more than one publication, sometimes in consecutive years. As a consequence, the number of all publications (n=26) documented here is larger than the total number of evaluation studies (n=19). Related publications carry the same ID and are distinguished by a, b, c (first row).
