# Supplementary material for: Professional standards in bibliometric research evaluation? A meta-evaluation of European assessment practice 2005–2019
Source: PLoS One. 2020 Apr 20;15(4):e0231735. doi: 10.1371/journal.pone.0231735 (PMC7170233; doi:10.1371/journal.pone.0231735)
Supplement: S8 Table — (DOCX) [file pone.0231735.s008.docx]

**S8 Table. Frequency of studies across countries and time periods**

| **Country** | **2005-2009** | **2010-2014** | **2015-2019** | **Studies total*** | **% studies** |
| --- | --- | --- | --- | --- | --- |
| Italy | 1 | 10 | 11 | 22 | 16 |
| Netherlands | 4 | 10 | 4 | 18 | 13 |
| United Kingdom | 1 | 7 | 8 | 16 | 12 |
| Sweden | 4 | 7 | 3 | 14 | 10 |
| Norway | 1 | 8 | 5 | 14 | 10 |
| Germany | 3 | 4 | 4 | 11 | 8 |
| Finland | 2 | 6 | 2 | 10 | 7 |
| European Union (ERA) | 2 | 5 | 4 | 9 | 7 |
| Spain | 2 | 3 | 3 | 8 | 6 |
| Denmark | 0 | 5 | 2 | 7 | 5 |
| Greece | 0 | 3 | 2 | 5 | 4 |
| Austria | 1 | 2 | 1 | 4 | 3 |
| Ireland | 0 | 1 | 2 | 3 | 2 |
| Switzerland | 1 | 0 | 2 | 3 | 2 |
| Hungary | 1 | 0 | 1 | 2 | 1 |
| Romania | 0 | 0 | 2 | 2 | 1 |
| Belgium | 0 | 1 | 0 | 1 | 1 |
| Island | 0 | 0 | 1 | 1 | 1 |
| Lithuania | 0 | 0 | 1 | 1 | 1 |
| Luxemburg | 0 | 1 | 0 | 1 | 1 |
| Serbia | 0 | 0 | 1 | 1 | 1 |
| Slovakia | 0 | 0 | 1 | 1 | 1 |
| Studies covering two or more countries | 2 | 2 | 3 | 7 | 5 |
| **Number of countries (+ EU)** | **11** | **14** | **19** | **21** | **­–** |
| **Studies total** | **21** | **64** | **53** | **138** | **100** |

Source: Meta-evaluation study set, 2005–2019

*Some studies cover evaluation objects from more than one country.
