# Supplementary material for: Professional standards in bibliometric research evaluation? A meta-evaluation of European assessment practice 2005–2019
Source: PLoS One. 2020 Apr 20;15(4):e0231735. doi: 10.1371/journal.pone.0231735 (PMC7170233; doi:10.1371/journal.pone.0231735)
Supplement: S9 Table — (DOCX) [file pone.0231735.s009.docx]

**S9 Table. Databases for bibliometric assessment and time periods**

| **Databases** | **% 2005-2009** | **% 2010-2014** | **% 2015-2019** | **% total** | **Studies total** |
| --- | --- | --- | --- | --- | --- |
| Web of Science (WoS) | 95 | 84 | 87 | 87 | 120 |
| – WoS improved versions | 48 | 44 | 53 | 48 | 66 |
| Scopus | 0 | 19 | 32 | 21 | 29 |
| Google Scholar | 0 | 9 | 8 | 7 | 10 |
| Disciplinary databases (e.g. PubMed) | 5 | 5 | 8 | 6 | 8 |
| National research databases (e.g. Cristin) | 10 | 22 | 32 | 24 | 33 |
| Organization-specific databases | 5 | 0 | 6 | 3 | 4 |
| Patent database | 5 | 0 | 0 | 1 | 1 |
| **Studies total** | **21** | **64** | **53** | **138** |  |

Source: Meta-evaluation study set, 2005-2019
