# Supplementary material for: Professional standards in bibliometric research evaluation? A meta-evaluation of European assessment practice 2005–2019
Source: PLoS One. 2020 Apr 20;15(4):e0231735. doi: 10.1371/journal.pone.0231735 (PMC7170233; doi:10.1371/journal.pone.0231735)
Supplement: S10 Table — (DOCX) [file pone.0231735.s010.docx]

**S10 Table. Enhanced data quality and time periods**

|  | **Dedicated organizations** | | | **Other bibliometric experts** | | |
| --- | --- | --- | --- | --- | --- | --- |
| **Improvement** | **% 2005-2009** | **% 2010-2014** | **% 2015-2019** | **% 2005-2009** | **% 2010-2014** | **% 2015-2019** |
| WoS improved versions | 71 | 73 | 60 | 36 | 10 | 17 |
| Institutional adresses cleaned | 86 | 89 | 100 | 43 | 30 | 74 |
| Author names disambiguated | 86 | 68 | 93 | 43 | 55 | 43 |
| Corrections for self-citations | 71 | 41 | 47 | 36 | 30 | 17 |
| Database coverage* | 71 | 70 | 53 | 29 | 25 | 4 |
| Validity of field definition** | 0 | 7 | 10 | 0 | 15 | 17 |
| Check of publication lists by authors | 43 | 9 | 3 | 29 | 35 | 4 |
| **Studies total** | **7** | **44** | **30** | **14** | **20** | **23** |

Source: Meta-evaluation study set, 2005-2019. * ‘Database coverage’ refers to analyses of internal or external coverage of scientific fields by citation databases. ** ‘Validity of field definition’ refers to the congruence between a bibliometric field definition and the targeted field of research.
