# Supplementary material for: Professional standards in bibliometric research evaluation? A meta-evaluation of European assessment practice 2005–2019
Source: PLoS One. 2020 Apr 20;15(4):e0231735. doi: 10.1371/journal.pone.0231735 (PMC7170233; doi:10.1371/journal.pone.0231735)
Supplement: S11 Table — (DOCX) [file pone.0231735.s011.docx]

**S11 Table. Frame of reference for research assessment and time periods**

|  | **International field comparison** | | | **National ranking** | | |
| --- | --- | --- | --- | --- | --- | --- |
| **Evaluation object** | **2005-2009** | **2010-2014** | **2015-2019** | **2005-2009** | **2010-2014** | **2015-2019** |
| % research organizations | 69 | 60 | 49 | 0 | 35 | 44 |
| % funding instruments | 60 | 90 | 90 | 0 | 0 | 0 |
| **% Studies analysed** | **67** | **70** | **57** | **0** | **23** | **36** |
| # research orgs. | 16 | 43 | 43 | 16 | 43 | 43 |
| # funding inst. | 5 | 21 | 10 | 5 | 21 | 10 |
| **# Studies total** | **21** | **64** | **53** | **21** | **64** | **53** |

Source: Meta-evaluation study set, 2005-2019
