# Supplementary material for: Professional standards in bibliometric research evaluation? A meta-evaluation of European assessment practice 2005–2019
Source: PLoS One. 2020 Apr 20;15(4):e0231735. doi: 10.1371/journal.pone.0231735 (PMC7170233; doi:10.1371/journal.pone.0231735)
Supplement: S12 Table — (DOCX) [file pone.0231735.s012.docx]

**S12 Table. Type of impact metrics and time periods**

|  | **Dedicated organizations** | | | **Other bibliometric experts** | | |
| --- | --- | --- | --- | --- | --- | --- |
| **Type of metric** | **% 2005-2009** | **% 2010-2014** | **% 2015-2019** | **% 2005-2009** | **% 2010-2014** | **% 2015-2019** |
| **Observed citation impact** | **86** | **100** | **100** | **50** | **90** | **91** |
| Field-normalized impact total | 71 | 98 | 97 | 43 | 35 | 39 |
| H-index and h-type indicators | 0 | 5 | 3 | 29 | 50 | 52 |
| Other observed impact | 100 | 82 | 90 | 21 | 40 | 52 |
| **Journal impact** | **29** | **48** | **50** | **57** | **55** | **43** |
| **Studies total** | **7** | **44** | **30** | **14** | **20** | **23** |

Source: Meta-evaluation study set, 2005-2019
