# Supplementary material for: Professional standards in bibliometric research evaluation? A meta-evaluation of European assessment practice 2005–2019
Source: PLoS One. 2020 Apr 20;15(4):e0231735. doi: 10.1371/journal.pone.0231735 (PMC7170233; doi:10.1371/journal.pone.0231735)
Supplement: S13 Table — (DOCX) [file pone.0231735.s013.docx]

**S14 Table. Classification of science for field normalization and time periods**

|  | **Dedicated organizations** | | | **Other bibliometric experts** | | |
| --- | --- | --- | --- | --- | --- | --- |
| **Classification** | **% 2005-2009** | **% 2010-2014** | **% 2015-2019** | **% 2005-2009** | **% 2010-2014** | **% 2015-2019** |
| Web of Science Classification | 86 | 89 | 67 | 64 | 35 | 30 |
| Scopus Classification | 0 | 11 | 37 | 0 | 5 | 9 |
| Essential Science Indicators | 0 | 5 | 0 | 7 | 5 | 4 |
| Alternative journal based classification* | 0 | 0 | 0 | 0 | 20 | 4 |
| Self-defined journal sets | 0 | 11 | 0 | 7 | 10 | 4 |
| Keywords combined with journal sets | 0 | 5 | 0 | 0 | 5 | 4 |
| Publication-based clusters | 0 | 0 | 23 | 0 | 0 | 0 |
| Other | 0 | 2 | 3 | 0 | 10 | 9 |
| **Studies with field normalization** | 86 | 100 | 100 | 50 | 50 | 48 |
| **Studies total** | **7** | **44** | **30** | **14** | **20** | **23** |

Source: Meta-evaluation study set, 2005-2019
