# Supplementary material for: Professional standards in bibliometric research evaluation? A meta-evaluation of European assessment practice 2005–2019
Source: PLoS One. 2020 Apr 20;15(4):e0231735. doi: 10.1371/journal.pone.0231735 (PMC7170233; doi:10.1371/journal.pone.0231735)
Supplement: S1 Appendix — (DOCX) [file pone.0231735.s014.docx]

**S8 Appendix. Meta-evaluation coding questions.**

Most items were coded in a standardized nominal format. Ordinal format was used where applicable. Items with a non-standardized format are marked as “open items”.

1. Study meta-information
   1. Who conducted the study? (open item)
   2. Is the evaluation object (EO) a research organization (RO) or funding instrument (FI)?
   3. When was the study published?
   4. In which country is the EO located?
   5. What is the publication format (journal article, report, etc.)?
2. Professional framework of bibliometric analysis
   1. Was the study conducted by experts internal or external to the EO?
   2. Were the authors working as members of a bibliometric expert organization or as individual bibliometric experts?
   3. Does the bibliometric assessment stand alone or constitute part of a broader evaluation, i.e. are bibliometric data combined with other data (e.g. peer review, financial data, or survey data)?
3. Object of the bibliometric evaluation
   1. What is the precise object of study (name and number of RO and sub-units, FI and units funded, individual scientists)?
   2. What is the main research question? (open item)
   3. What are the main variables of the bibliometric analysis? (open item)
   4. Does the study include a treatment comparison (treatment vs. control group design)?
   5. Does the study contain a bibliometric collaboration analysis?
4. Bibliometric databases
   1. Which databases or data sources are used for the bibliometric assessment?
   2. Which document types are included in the analysis?
5. Quality enhancement of bibliometric raw data

Does the study contain information regarding the following:

- 1. the cleaning of author names?
  2. the cleaning of institutional addresses?
  3. the congruence of the research field(s) under study with the bibliometric field delineation?
  4. the coverage of the research field(s) under study by the utilized database?
  5. the personal control of publication lists by authors subject to evaluation?

1. Sampling strategy and data collection
   1. What sampling strategy is used for data collection?
   2. Is sampling exclusively based on information in citation databases or did the organization under evaluation provide publication lists?
   3. What is the time period of publication sampling?
   4. Does the study contain time series data?
   5. What is the total number of publications in the sample?
2. Research fields under evaluation
   1. Which research fields are being assessed? (open item)
   2. Do the research fields belong to disciplines that are well covered (or less well covered) by the Web of Science (WoS), based on Moed (2005) and information in Table 3 (main document)?
3. Definition of citation data
   1. Does the analysis consider self-citations?
   2. Does the analysis use whole and/or fractional citation counts?
   3. Does the analysis distinguish author positions (first authors)?
   4. Does the study use fixed citation windows or cumulative citation counts?
   5. How long are fixed citation windows?
4. Citation impact indicators
   1. Which type of citation metrics are employed: field-normalized total; journal impact metrics; field-normalized arithmetic mean; other field percentiles; indirect impact metrics; source-normalized metrics; h-index and h-type indices; other impact metrics?
   2. How exactly are citation impact metrics defined? (open item)
   3. Which classification of science fields is used for field normalization?
5. Statistical methods used
   1. Does the study contain statistical tests?
   2. Does the study offer any special methodological features, including methods of analysis or software? (open item)
